# Flow Biocatalysis: A Challenging Alternative for the Synthesis of APIs and Natural Compounds

**DOI:** 10.3390/ijms22030990

**Published:** 2021-01-20

**Authors:** Micol Santi, Luca Sancineto, Vanessa Nascimento, Juliano Braun Azeredo, Erika V. M. Orozco, Leandro H. Andrade, Harald Gröger, Claudio Santi

**Affiliations:** 1 Chair of Industrial Organic Chemistry and Biotechnology, Faculty of Chemistry, Bielefeld University, Universitätsstraße 25, 33615 Bielefeld, Germany; harald.groeger@uni-bielefeld.de; 2 Group of Catalysis Synthesis and Organic Green Chemistry, Department of Pharmaceutical Sciences, University of Perugia Via del Liceo 1, 06100 Perugia, Italy; luca.sancineto@unipg.it; 3 Department of Organic Chemistry, Institute of Chemistry, Universidade Federal Fluminense, Valonguinho Campus, Niterói 24020-141, Brazil; nascimentovanessa@id.uff.br; 4 Departament of Pharmacutical Science, UNIPAMPA, BR 472-KM 585, Uruguaiana 97501-970, Brazil; julianoazeredo@unipampa.edu.br; 5 Institute of Chemistry, University of São Paulo, Av. Prof. Lineu Prestes 748, São Paulo 05508-900, Brazil; erikamenaca@usp.br (E.V.M.O.); leandroh@iq.usp.br (L.H.A.)

**Keywords:** biocatalysis, flow chemistry, immobilized enzyme, packed-bed reactor, segmented flow, microreactors, APIs, natural products, sustainability

## Abstract

Biocatalysts represent an efficient, highly selective and greener alternative to metal catalysts in both industry and academia. In the last two decades, the interest in biocatalytic transformations has increased due to an urgent need for more sustainable industrial processes that comply with the principles of green chemistry. Thanks to the recent advances in biotechnologies, protein engineering and the Nobel prize awarded concept of direct enzymatic evolution, the synthetic enzymatic toolbox has expanded significantly. In particular, the implementation of biocatalysts in continuous flow systems has attracted much attention, especially from industry. The advantages of flow chemistry enable biosynthesis to overcome well-known limitations of “classic” enzymatic catalysis, such as time-consuming work-ups and enzyme inhibition, as well as difficult scale-up and process intensifications. Moreover, continuous flow biocatalysis provides access to practical, economical and more sustainable synthetic pathways, an important aspect for the future of pharmaceutical companies if they want to compete in the market while complying with European Medicines Agency (EMA), Food and Drug Administration (FDA) and green chemistry requirements. This review focuses on the most recent advances in the use of flow biocatalysis for the synthesis of active pharmaceutical ingredients (APIs), pharmaceuticals and natural products, and the advantages and limitations are discussed.

## 1. Introduction

The term “biocatalysis” refers to the use of enzymes, both as purified proteins or as whole cells, to catalyze the conversion of a substrate into a desired product, and it is currently a popular topic in synthetic research [1]. Since 1858, when Louis Pasteur reported the kinetic resolution of racemic tartaric acid through fermentation promoted by microorganisms [2], enzymes have been widely studied and exploited to catalyze organic chemical transformations, especially in the food industry [3]. An awakening of the interest in biocatalytic applications has since occurred in the latter half of the 20th century [4], motivated by the need for greener and more sustainable chemical processes [5,6].

Since the advent of the environmental movement [5,6,7,8] and the definition of green chemistry and its 12 principles [9], chemical industries have undergone radical changes by investing in more sustainable methodologies to minimize wastes and their ecological footprints. As a result, biocatalysis has begun to receive considerable attention from the chemical industry, thanks to its greener and sustainable features, which fulfill 10 out of the 12 principles of green chemistry [10,11]. The major advantage of enzymatic catalysis is its ability to promote highly chemo-, regio- and stereoselective transformations. Over thousands of years of evolution, nature has equipped cells with highly efficient and extremely selective catalytic systems to promote the synthesis of every kind of natural product while reducing waste generation. Moreover, “natural” enzymes have also been found to catalyze “non-natural” synthetic reactions and are capable of increasing their stability by direct evolution to better adapt themselves to “non-natural” reaction conditions [12,13,14,15]. This higher selectivity leads to more straightforward synthetic pathways with no functionalization steps needed, resulting in reduced wastes [16,17]. Additionally, enzymes are biodegradable compounds (proteins) produced by renewable resources such as plants [18] or microorganisms [19], and work under mild conditions, such as relatively low temperatures, atmospheric pressure and in non-toxic, inexpensive, and readily available solvents, such as water.

The abovementioned benefits have contributed to the industrial use of biocatalysts for the synthesis of commercially interesting compounds such as pharmaceuticals or fine chemicals [20,21,22,23,24], aiming to satisfy economic demands while respecting the green chemistry requirements [25]. Furthermore, modern advancements in biotechnology [26] and immobilization techniques [27,28], as well as recent developments in protein engineering and direct evolution [12,13,14,15,29], have helped to enhance the utilization of biocatalysis in synthesis [30] by enabling cheaper production of enzymes and providing access to a large library of versatile biocatalysts.

Nevertheless, the number of industrial biocatalytic applications remains limited by difficulties in scaling up, tedious optimization processes and long reaction times, as well as enzyme instability over time and their incompatibility with other reaction conditions [1,4]. This incompatibility makes one-pot cascade reactions hard to perform, resulting in downstream purifications, which are usually time-consuming due to protein aggregations [31]. Moreover, the use of water as a sole solvent often leads to overly diluted solutions due to substrate solubility issues, resulting in high solvent usage, low product concentrations producing low productivities, as well as high energy-consumption due to the high boiling point required [4].

**Figure 1:**
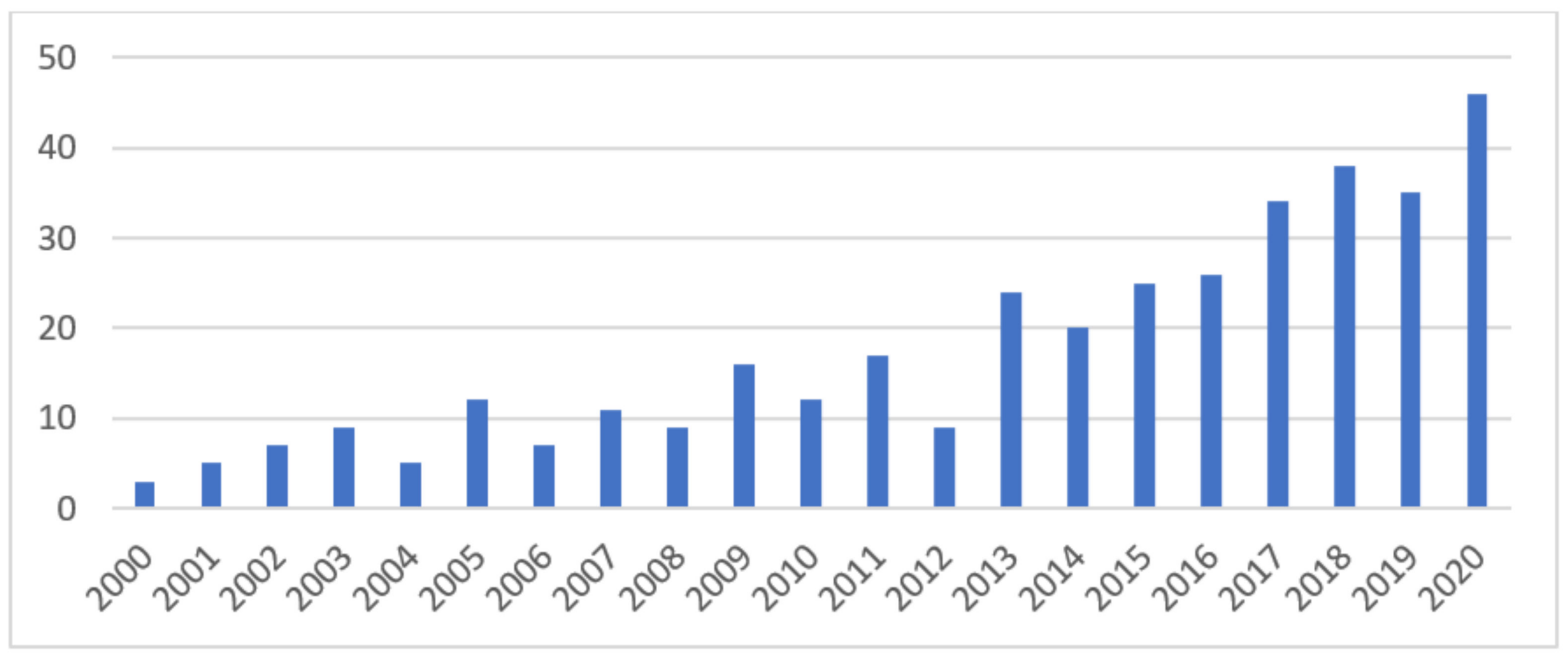
Overview of the number of flow biocatalysis-related publications added to SciFinder^®^ in the past 20 years.

A practical improvement in performances was reached with the advent of continuous-flow technology and the implementation of enzymatic reactions with flow reactors [31] and was also promoted by the arrival of several commercially available tools [32,33]. The rapid growth of interest in continuous-flow biocatalysis from both academia and industry is evidenced by the rising number of publications reporting it in the past decades, as depicted in Figure 1.

This increased interest in flow biocatalysis arose from the possibility of merging the conveniences of enabling technologies and the advantages of enzymatic catalysis into versatile, greener and sustainable synthetic tools. The ongoing evolution of this research area has led to the generation of numerous comprehensive review articles [34,35,36,37,38,39], which aimed to clarify the terminology as well as discuss the state-of-the-art and future perspectives of continuous-flow biocatalysis.

This review does not claim to be an exhaustive overview of all aspects of continuous flow biocatalysis but is more of an “add-on” to the existing review literature. The presented article, inspired by the opinion recently published by Martin et al. from Novartis [40], aims to complete the picture of modern “flow biocatalysis” by presenting recent applications in the synthesis of active pharmaceutical ingredients (APIs), natural products and key precursors for pharmaceutical production, highlighting its importance in the future of the pharmaceutical industry. Other aspects recently included in comprehensive reviews reported by De Santis et al. [34] and Britton et al. [36], as well as the catalysts special issue “Flow Biocatalysis” edited by Professor Paradisi [39], will be not discussed in the present article.

## 2. Biocatalysis in Industry

**Scheme 1:**
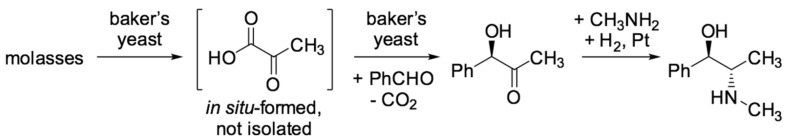
Pioneer work on industrial biocatalysis: chemoenzymatic production of l-ephedrine.

It is noteworthy that biocatalysis has not only gained interest in academia but has already found numerous applications over the last decades in the chemical industry [23,41,42]. In particular, production processes have been established for the large-scale manufacturing of chiral compounds that are needed as fine chemicals and pharmaceuticals [23,42]. To the best of our knowledge, the first industrial example dates back to the 1930s, with the technical chemoenzymatic process for l-ephedrine developed at Knoll AG, which was based on an initial “umpolung” reaction with a ligase and subsequent metal-catalyzed diastereoselective reduction (Scheme 1) [43]. Since then, there has been a dramatic increase in industrial examples over the past three to four decades [23,41,42]. Certainly, this field of industrial biocatalysis has strongly benefited from the impressive progress in molecular biology, which enabled the rapid location of suitable enzyme candidates in nature as well as an efficient production through tailor-made recombinant strains. Furthermore, various techniques in protein engineering have also enabled an effective improvement of the active sites of enzymes, addressing specific needs of the target reaction in terms of selectivity and activity, for example [44].

As previously mentioned, the high enantioselectivities achieved by enzymes in many transformations represent one of the key advantages of their use in the production of particular chiral molecules. In addition to the tremendous benefit of achieving excellent selectivities, further advantages are the mild reaction conditions and the outstanding economic performance data. The last criteria might sound surprising since enzymes are expected to represent a severe cost factor. However, when realizing access to a recombinant form of a biocatalyst (which today is the “standard form” of a biocatalyst), this type of catalyst can be produced in a highly cost-efficient way by means of high-cell density fermentation [45].

**Scheme 2:**
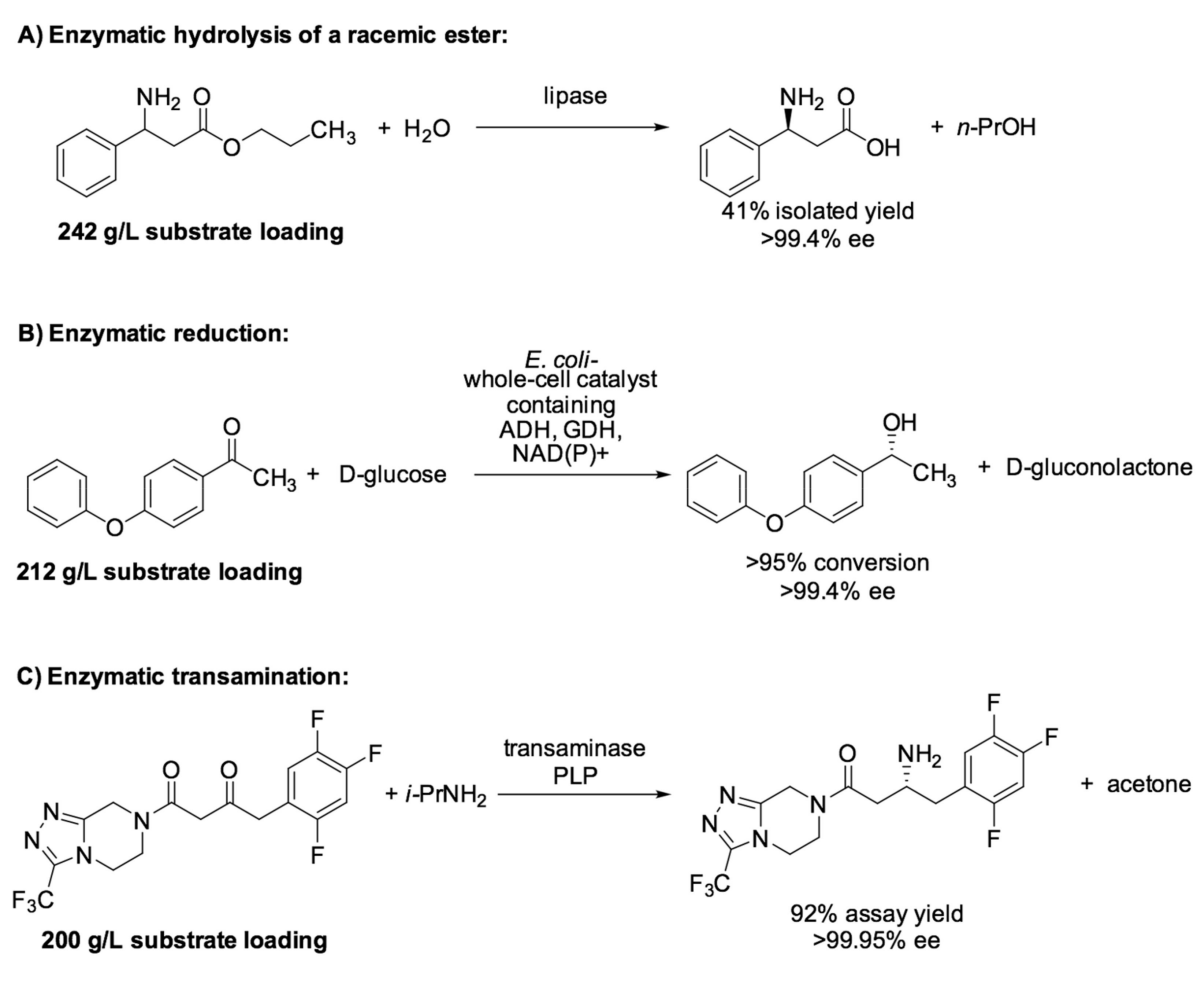
Representative examples for biocatalytic processes with different enzyme classes running at high substrate loading.

Furthermore, biotransformations can also be conducted under conditions required for technical purposes, such as at high substrate loading of 1 M, or even above, thus enabling excellent space–time yield data. Representatives recently reported examples of highly efficient biotransformations running at high substrate loadings of at least 200 g/L (shown in Scheme 2) were the lipase-catalyzed resolution of α-amino acid ester resolution (with a substrate loading of 242 g/L) [46] and the alcohol dehydrogenase-catalyzed enantioselective reduction of ketones (with a substrate loading of up to 212 g/L) [47], both reported by researchers from Degussa (now: Evonik Industries). An additional example was the transaminase-catalyzed enantioselective reductive amination of a ketone (with a substrate loading up to 200 g/L), reported jointly by researchers from Merck and Codexis [48].

**Scheme 3:**
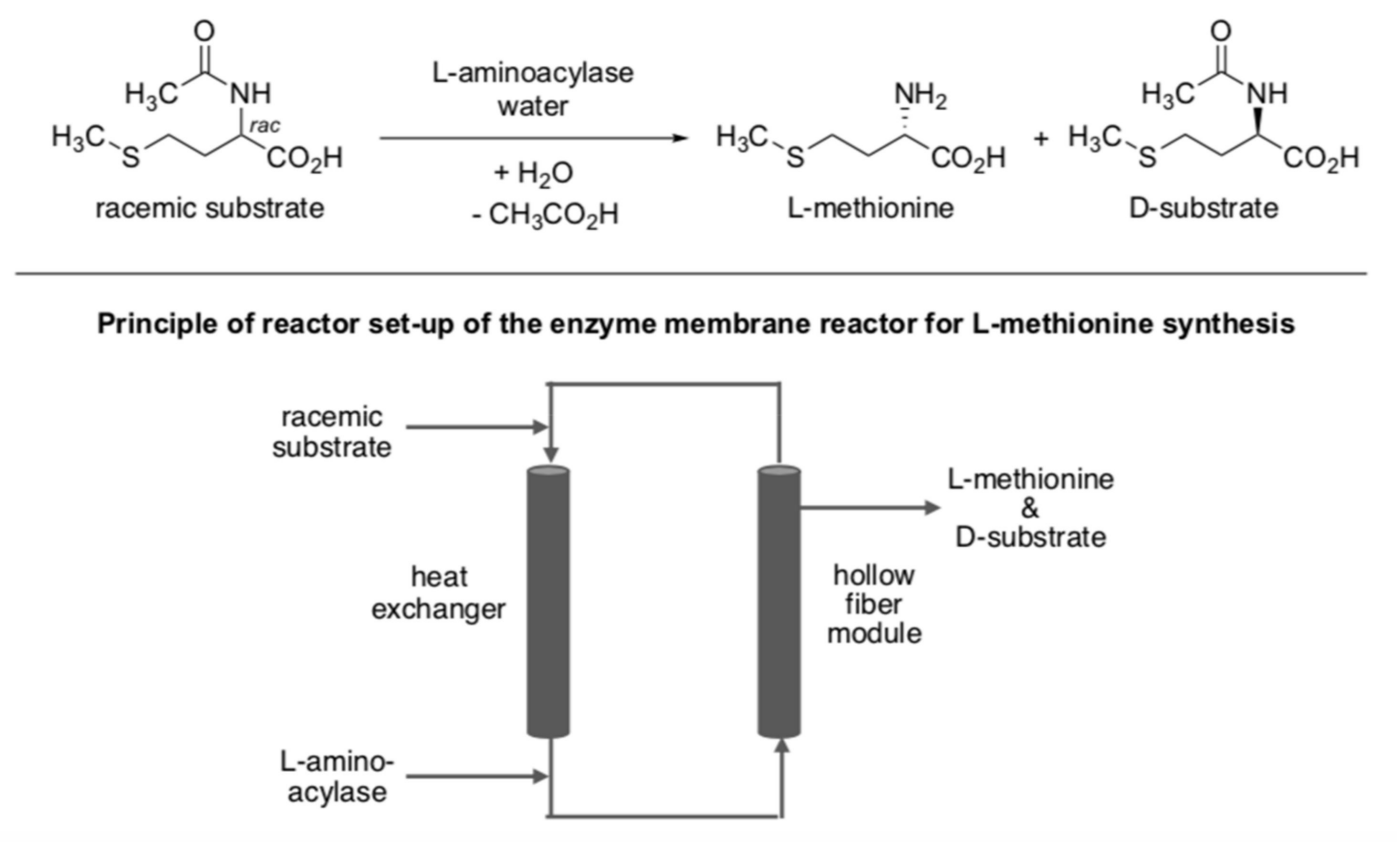
Continuously running enzymatic process for the production of l-methionine.

The majority of these reported processes are batch processes, and continuously running processes are still rare [23,42]. One of the exceptions is the aminoacylase-catalyzed resolution for the production of l-methionine on a large scale by means of an enzyme membrane reactor (Scheme 3) [49].

In spite of impressive production processes, however, a range of challenges arise from such batch transformations. In general, a widely known issue is related to the phase separation of biphasic media when utilizing larger amounts of proteins due to their emulsifying properties (e.g., when the enzymes are used as crude extract containing a large amount of further, non-needed proteins) [50]. Another challenge is mass transfer, as the mixing efficiency in batch media is limited despite the high stirring rates. Furthermore, enzyme deactivation in batch can be severe due to, for example, mechanical shear forces. A further general drawback of batches is, by definition, the different product qualities, as every batch typically gives a slightly different product quality.

This challenge of ensuring constant product quality has been a major driving force by institutions for drug approval such as the Food and Drug Administration (FDA) in the United States as well as the European Medicines Agency (EMA) in Europe to encourage pharmaceutical producers in their efforts to switch from batch to flow mode [51,52,53]. Furthermore, the opportunities of flow processes can also address further challenges in batch processes, such as mass transfer, avoidance of shear forces and avoidance of emulsion formation. Thus, flow processes with enzymes can contribute to increase the enzyme’s stability and lifetime, while at the same time, simple downstream-processing can be realized due to the lack of emulsion formation, thus leading to an efficient phase separation. Note that work-up often has a significant impact on production costs, and decreasing the number of unit operation steps during downstream processing can significantly improve economic data.

Thus, there has also been an increasing interest in realizing biocatalytic transformations in flow mode in response to a growing demand for greener yet highly selective and efficient synthetic processes.

## 3. Fundamentals of Flow Biocatalysis

**Figure 2:**
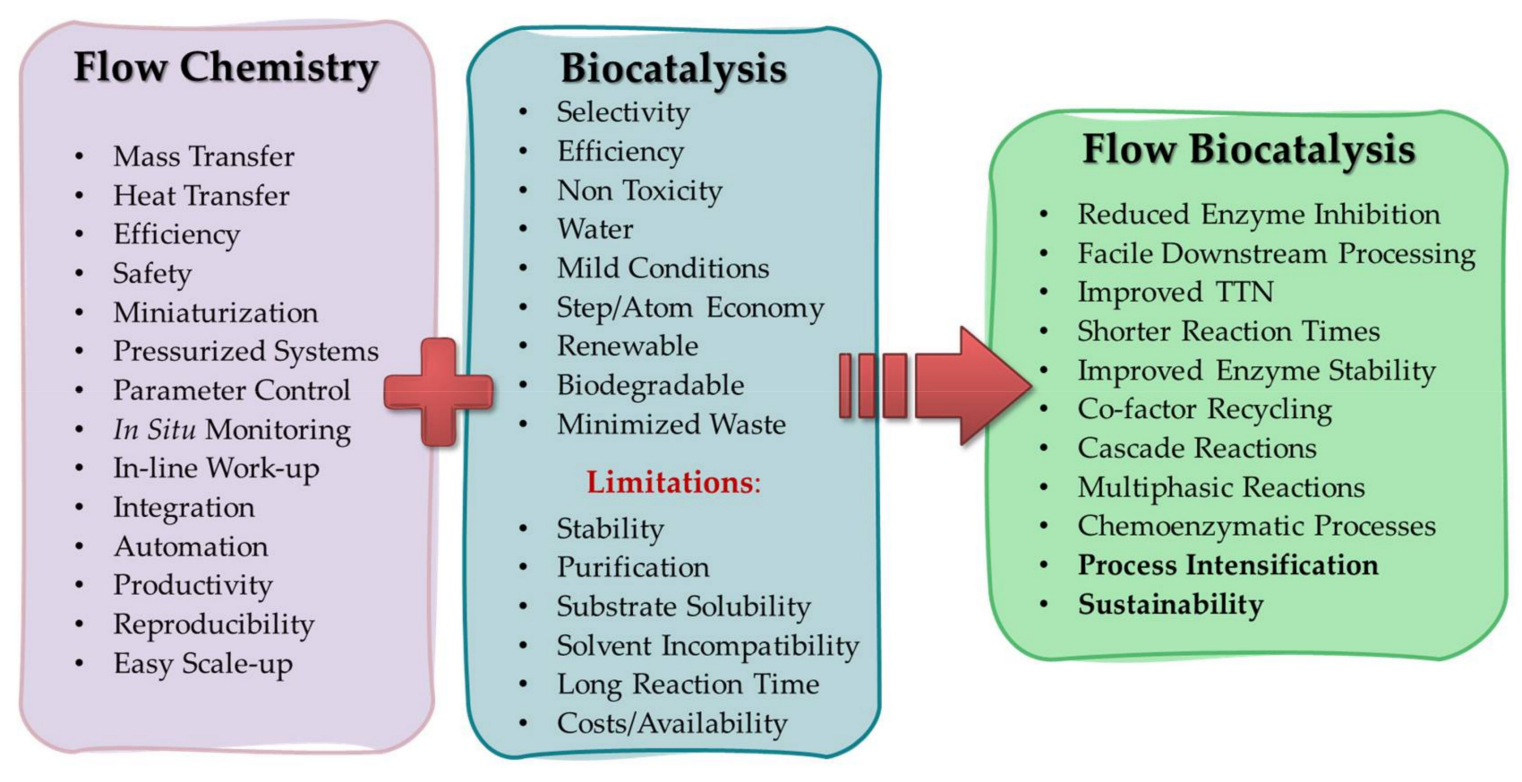
Overview of the key advantages of flow chemistry, biocatalysis and their benefits in flow biocatalysis; TTN = Total turnover number.

Continuous flow technology has been around for the past 30 years [54,55] and has found numerous applications in synthesis [32,56,57,58], especially in the synthesis of pharmaceuticals [24,59] and fine chemicals [21]. When performing a reaction in a flow system, the solutions containing the reagents are pumped through channels and/or tubes, where they mix and react in a continuous stream instead of inside a flask, and product formation increases along the length of the reactor instead of with time [32,60]. This unconventional technique offers several advantages (Figure 2), such as improved mass and heat transfer, better control over reaction conditions and better handling of hazardous compounds [61,62,63]. In particular, multiphasic reactions benefit from fluidic systems such as miniaturized reactor or segmented flow, in which the increased surface to volume ratios and interphases play important roles in providing efficient mixing [32].

For instance, gaseous reagents often present poor interfacial mixing due to low solubility, leading to long reaction times. This issue can be avoided by using pressurized flow reactors in which the gas solubility is increased, and the gas flow is handled in a safer and more controlled manner [64]. Another key advantage of flow chemistry is its easy integration with complementary enabling technology, such as electrochemical, photochemical or microwave units, as well as with inline quenching units and in/online analytical units for real-time monitoring in situ and multistep processes. Importantly, the introduction of inline and online analytic devices has shifted the attention of researchers towards automation [33,65] and the employment of automated feedback optimization [66,67], which has proven to be highly effective for high-throughput screening (HTS) and process optimization. All the abovementioned features ensure reproducibility, scalability and productivity, and explain why the area of flow chemistry has achieved great success, especially in synthesis, and why it is increasingly applied in the industrial environment [68,69,70].

The field of biocatalysis itself is embracing the benefits of flow chemistry to overcome limitations commonly faced with “classic” enzymatic catalysis such as solubility issues, enzyme inhibition and difficult downstream purifications. One of the earliest reported and most popular methods of flow biocatalysis is the use of immobilized enzymes in packed-bed reactors (PBRs). Enzyme immobilization has already been proven to improve the stability and selectivity of the enzyme [71], and the implementation of continuous flow systems simplifies product purification through enzyme retention. Additionally, the continuous removal of product and substrate in flow reduces enzyme inhibition, improving the total turnover number (TTN) of the enzyme, hence the reaction rates [21]. The cofactor regeneration often represents the rate-determining step of the catalytic cycle. However, the integration of flow reactors with electrolytic methods was shown to speed up the in situ electrochemical regeneration of cofactors such as NAD^+^ and NADH [72,73]. Furthermore, the reaction rate of biocatalytic oxidations, which relies on poorly water-soluble molecular oxygen gas, could be remarkably improved by performing the reactions in pressurized continuous flow systems. As previously mentioned, pressurized systems have been helpful in increasing the concentration of a dissolved gas, and studies showed that a threefold increase in the oxygen content positively affected the enzyme efficacy [74]. Besides single-step transformations, continuous-flow systems can facilitate the performance of enzymatic cascade reactions by combining more reactors in series. Additionally, this technology is particularly useful for the combination of chemo- and biocatalysis for multistep syntheses [75]. Chemoenzymatic processes are characterized by numerous advantages, such as higher yields and selectivity, as well as minimized costs and wastes, with obvious environmental benefits. However, the combination of both “catalytic worlds” is limited by incompatible divergent reaction conditions, such as reagent and solvent tolerance. Nevertheless, continuous flow systems can enable chemoenzymatic processes by special separation [21,76,77].

**Figure 3:**
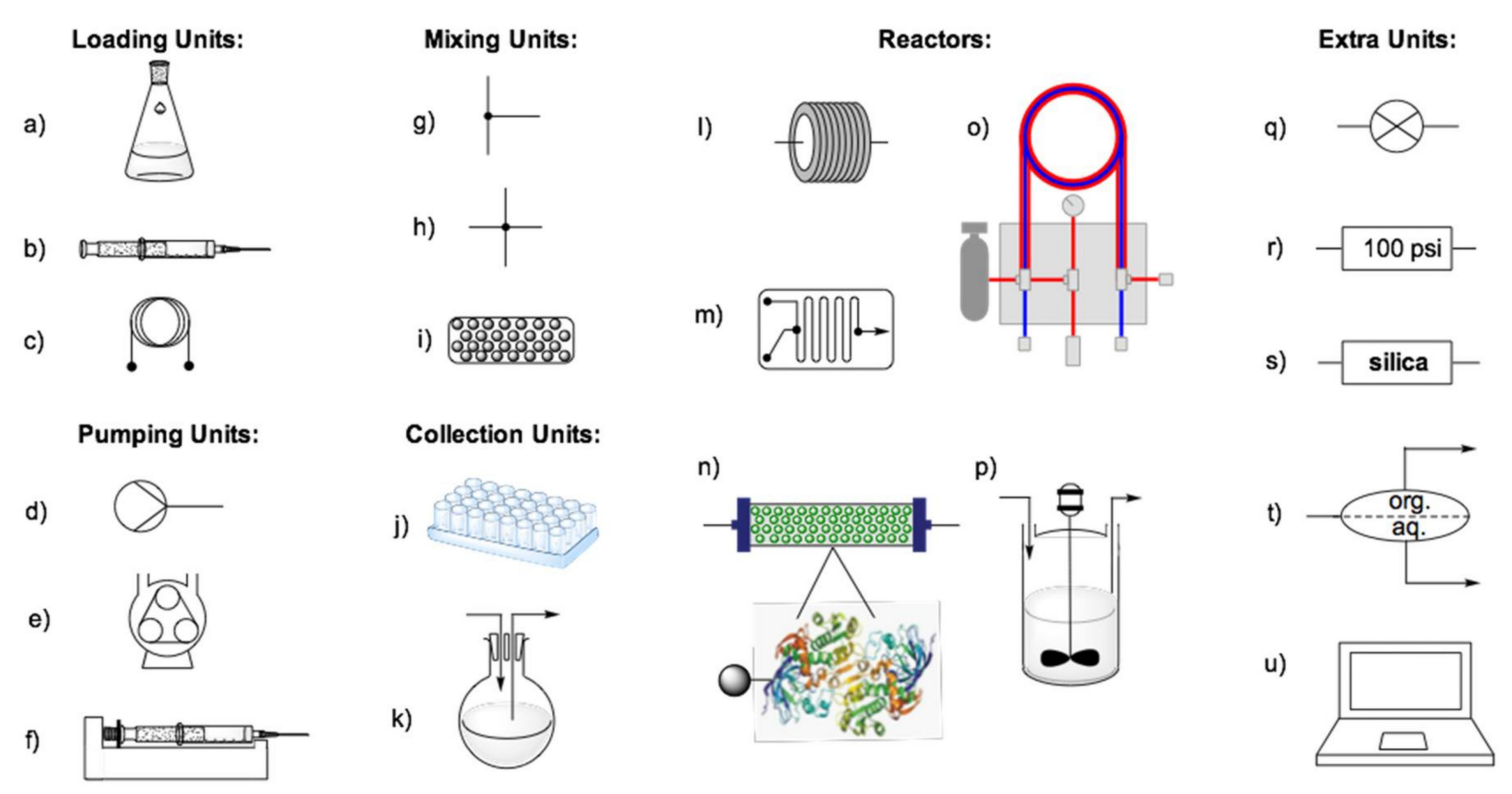
Basic continuous flow equipment: (**a**) reservoir; (**b**) syringe; (**c**) sample loop; (**d**) general pump; (**e**) peristaltic pump as an example of continuous pumps; (**f**) syringe pump as an example of semicontinuous pumps; (**g**) T-piece mixer; (**h**) quad mixer; (**i**) static mixer; (**j**) fraction collector; (**k**) inline collection flask; (**l**) coil reactor; (**m**) microreactor; (**n**) packed-bed reactor (PBR); (**o**) tube-in-tube reactor; (**p**)continuous-stirred tank reactor (CSTR); (**q**) inline valve; (**r**) back pressure regulator (BPR); (**s**) inline silica plug; (**t**) inline liquid–liquid phase separator; (**u**) realtime reaction monitoring.

Continuous flow biocatalysis provides access to new, greener and more efficient synthetic possibilities, and in the last five years, there has been a rapid growth in the number of techniques and methodologies, which have also been promoted by the high availability of commercial apparatus. A typical continuous flow system is composed of a sampling unit, such as a reservoir, a syringe or a sampling loop, a pumping unit, a mixing unit, a reactor and a collection unit (Figure 3). Additionally, extra modules, such as a back pressure regulator (BPR) and an inline phase separator, can be used to pressurize the system or perform downstream extraction in flow. Finally, inline switching valves and inline analytical cells can be added to monitor the reactions in real-time [32,36].

In a laboratory-scale environment, flow bioreactors can be classified into two main classes: microreactors and mesoreactors [37]. The microfluidic devices are characterized by internal diameters between 10 and 500 µm and are usually either microchip or microcapillary reactors [78,79]. On the other hand, mesoreactors present channels with internal diameters from 500 µm up to a few millimeters. Although microreactors enhance efficient mixing and enable laminar flow and accurate temperature control, they are easier to block and represent a more expensive choice due to their glass manufacturing. For these reasons, mesoreactors, such as PTFE of FEP coils, glass packed-bed reactors or wall-coated reactors for immobilized enzymes, are more popular. Membrane reactors such as the tube-in-tube device, which is used for biotransformations that involve gaseous reagents, are another type of mesoreactor [80,81]. More recently, 3D-printed continuous flow reactors have also begun to be applied to biocatalysis to create customizable bioreactors [82,83,84,85]. The characteristics of the abovementioned flow bioreactors have been extensively reviewed recently [34,36,39].

Either biotransformations catalyzed by enzymes from cell-free systems or whole cells can be translated into flow mode. Isolated enzymes, obtained via overexpression in a host microorganism [86,87,88], are very popular and often commercially available since high selectivity and fast reaction rates can be achieved. This is due to the higher concentration of enzymes compared to the same mass of the whole cells and the fact that substrates can easily reach active sites with no cell-membrane penetration needed [89,90]. However, cell-free enzymatic reactions present several disadvantages, such as the costs of enzyme purification. Additionally, some cofactor-dependent enzymes require the external addition of cofactors. Moreover, some purified enzymes may be less stable outside their native medium. On the other hand, whole-cell mediated reactions are less expensive, and enzymes operate within their native environment; thus, good stability can be expected and there is no need for external addition of cofactors [91]. However, they demonstrate slower reaction rates as they are limited by membrane penetration of the substrate [92,93], and because the reaction takes place within the whole cell complexity, secondary metabolic pathways may lead to undesired side products [36].

**Figure 4:**
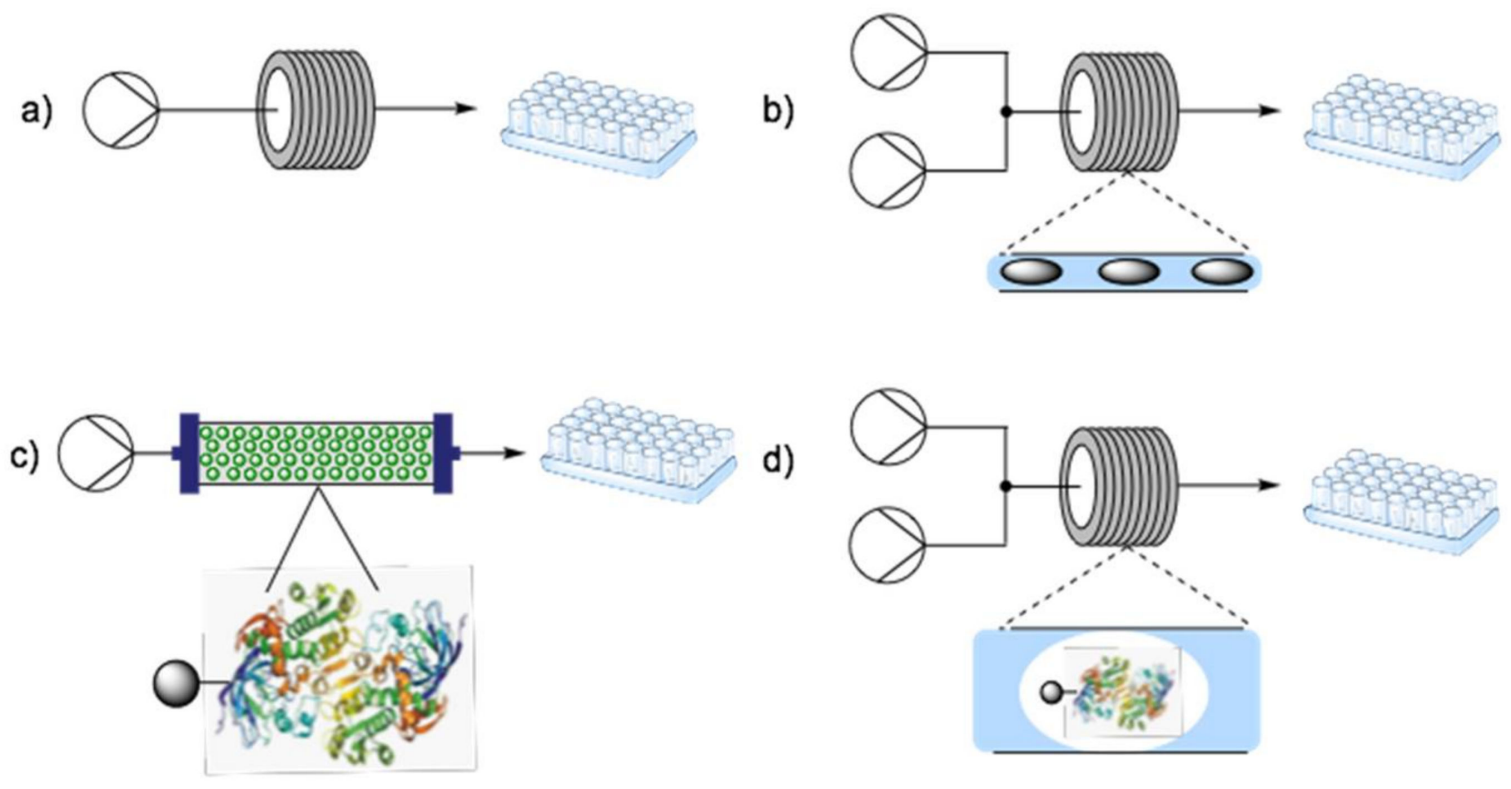
Examples of simple flow biocatalytic setups: (**a**) monophasic; (**b**) biphasic segmented flow; (**c**) immobilized enzyme of PBR; (**d**) catalytic hydrogel segmented flow.

Both types of biocatalysts can be used in buffer solutions (Figure 4). In the simplest bioreactor, the enzyme and substrate are simultaneously pumped through the reactor (a). Alternatively, biphasic systems, such as parallel laminar flow or segmented flow regimes (b), can be used when handling water-insoluble substrates/products in order to prevent precipitate formation, which will create blockages within the channels [94]. However, to facilitate downstream processing and product purification, as well as improve reaction rates and enzyme stability [71], the immobilization on solid supports (c) is usually preferred [27].

A carrier should be easy to regenerate and should present a large surface bearing functional groups for the enzyme attachment [95]. Moreover, it should ideally be rigid, thermally stable, chemically inert, water-insoluble, resistant toward microbial degradation and nontoxic [96]. Examples of such supports are cellulose [97], coconut fibers [98], kaolin [99], molecular sieves [100], silica [101] and agarose [102], which have been utilized in several different physical forms such as monoliths [103], supermagnetic nanoparticles [28,104,105] and hydrogels [106,107,108]. In wall-coated reactors, the enzyme can be immobilized directly on the reactor’s walls or on a membrane [109,110,111], whereas in PBR, the supported-enzyme particles are packed in a glass column. It is noteworthy that, in the last year, the first application of “fluid” immobilized enzymes in continuous flow processes was reported [112]. In this case, heterogeneous biocatalysts were used as the mobile hydrogel phase along with an organic solvent in a segmented flow process (Figure 4d). There are four main methods to immobilize biocatalysts onto solid carriers: by adsorption, by entrapment, by affinity and by covalent bonding. Carrier-free immobilization is also possible via crosslinking between surface NH_2_ groups leading to crosslinked enzyme crystals (CLECs) or crosslinked enzyme aggregates (CLEASs) upon crystallization or precipitation, respectively [27].

The selection of the right immobilization method is crucial and can influence the enzyme activity since the interaction between the enzyme and support can affect its tridimensional structure. There is not one preferred immobilization method for all types of enzymes as each molecule presents a unique and complex protein structure. Hence, factors such as catalytic activity, enzyme deactivation and enzyme leaching, as well as costs for immobilization, must be taken into consideration [113].

When translating a biotransformation from a batch to a flow system, there are several parameters that need to be considered. Even for simple monophasic systems (Figure 4a), the reaction rate will depend on the residence time as well as on the enzyme kinetics [114]. The residence time (*τ*), which is the time the reactants spend inside the reactor, is calculated from the reactor volume (*V*) and the volumetric flow rate (*Q*) via the simple equation:

(1) $$\tau =\frac{V}{Q}$$

It is worth mentioning that, in an ideal plug-flow system, each bioreactor will be characterized by a well-defined residence time. However, in real-life, multiple *τ*’s exist at the same time as a consequence of the parabolic flow due to frictions with the reactor walls, hence the discussion of residence time distribution in which there will be an “equilibration” period before a steady state is reached. This aspect is important to consider when calculating yields and performing multistep synthesis.

Moreover, the productivity of an enzymatic reaction is often expressed by the specific reaction rate (*r*), which is the amount of product that can be formed in 1 min by 1 g of enzyme. The specific reaction rate in a continuous-flow reaction (*r_flow_*) can be calculated as [115]:

(2) $$r_{flow}=\frac{\left[P\right]\times Q}{m_{e}}$$

where [*P*] is the product concentration in µmol/mL, *Q* is the volumetric flow rate in mL/min and *m_e_* in the catalyst loading (g), which can be alternatively substituted by the unit of enzyme activity “U” and, in this case, will be expressed in mg. On the other hand, for a biotransformation in a stirred batch reactor, the specific reaction rate (*r_batch_*) is calculated from the amount of product [*P*] (µmol/mL), the reaction time *t* (min) and the mass of the applied enzyme *m_e_*:

(3) $$r_{batch}=\frac{\left[P\right]}{t\times m_{e}}$$

Therefore, a direct comparison between batch and flow reaction rates of biocatalyzed processes can only be made at the same degree of conversions since the product(s) formation does not follow the same kinetics in the two different conditions.

Furthermore, in the translation of a biotransformation in a flow mode, besides the more classic parameters such as substrate concentration, biocatalyst loading and activity, other important parameters that are directly related to the reactor need to be considered. For instance, the reactor volume and internal diameter, as well as the void volume for packed-bed and monolith reactors. The drop in pressure between the two ends of a PBR should also be taken into account, especially when multiple steps are combined in series.

Finally, the stability of the reactor over time, the biocatalyst productivity (expressed as the total turnover number (TTN) and indicating the product synthesized per amount of used enzyme) as well as the reactor productivity (reported as the space/time yield normalized by the reactor volume) needs to be indicated to fully characterize an enzymatic flow reaction and its efficiency.

## 4. Flow Biocatalysis Applied to the Synthesis of Active Pharmaceutical Ingredients (APIs) and Their Precursors

Nowadays, the pharmaceutical industry is starting to invest more into continuous manufacturing as a valuable alternative to conventional batch processing to build good manufacturing practice (GMP) facilities [40]. In the following section, recent examples of flow biocatalysis applied to the synthesis of pharmaceutically interesting compounds or precursors are described. Firstly, the advantages and recent significant developments of specific methodologies are presented in single-step processes. Subsequently, the power of continuous flow implementation in biocatalysis is further demonstrated by a few examples of multi-step synthesis, including large scale synthesis, which represent a possible alternative that industry could adopt to move towards a more sustainable future.

### 4.1. Single-Step Flow Biocatalytic Systems

**Scheme 4:**
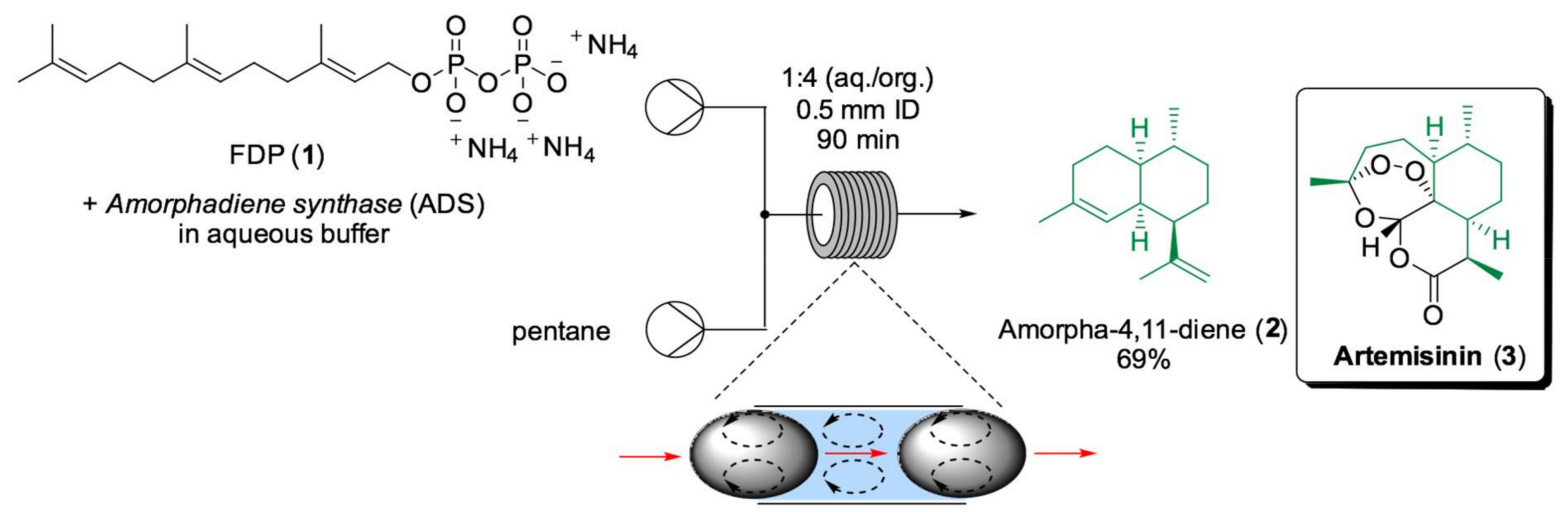
Amorpha-4,11-diene (**2**) biosynthesis in segmented flow. The red arrows show the direction of the flow, while the black arrows represent the convective motions [122].

One common rate-limiting step of enzymatic reactions is the slow release of the hydrophobic product into the aqueous medium. To solve this problem, an organic/water biphasic system could be used in which the hydrophobic product is continuously extracted from the aqueous phase by an organic solvent; thus, the reaction equilibrium could be shifted towards product formation. For instance, the efficiency of terpene synthases for the synthesis of sesquiterpenes is compromised by slow product release, which causes the enzyme to stall during aqueous medium saturation due to the low solubility of sesquiterpenes in water [116]. Sesquiterpenes are a large class of natural products that present pharmaceutical and agricultural properties [117]. This family of compounds is characterized by a complex structure and molecular formula C_15_H_24_, which can also present several stereogenic centers that make total synthesis challenging and low yield [118,119]. Considering their interesting biologically active properties, finding an efficient and selective synthetic route towards sesquiterpenes is highly desirable. For example, amorpha-4,11-diene (**2**), a key intermediate in the synthesis of the antimalarial drug artemisinin (**3**), can be obtained from the conversion of linear precursor farnesyl diphosphate (FDP) (**1**), catalyzed by amorphadiene synthase (ADS) [120]. The ADS generates the polycyclic core of amorpha-4,11-diene (**2**) with excellent regio- and stereocontrol; however, its applications remain limited by ADS sensitivity to heat and acidic conditions, as well as the insolubility of **2** in water. Traditional, two-phase systems in batch suffer from low mass transfer due to the minimal area of interface between the two immiscible solvents and from the deactivation of the enzyme through long exposure to organic solvents [121]. As a solution to these limitations, the Allemann and Wirth groups reported the use of several sesquiterpene synthases in segmented flow systems to accelerate mixing and shorten reaction times (Scheme 4) [122].

In a segmented flow system, the interfacial area between the two liquids is increased and controlled by the size of the solvent segments [123]. The system design for enhanced terpene synthase catalysis is composed of two immiscible liquids, water and pentane, pumped using syringe pumps through a T-mixer and a 2 mL coil reactor. The resulting system is a segmented flow with a higher surface-to-liquid volume ratio, hence a better mass-transfer rate. Note that, due to the presence of shear forces between the tubing wall and the axis of the flow, convective motions inside each segment ensure the renewal of the interfacial area, facilitating product extraction into the organic phase [124]. The reaction mixture is then collected in a flask, and the two phases are separated by gravity. For the preliminary studies, aristolochene synthase (AS) was used as a model enzyme [125] in a premixed solution with FDP (**1**) in a pH 7.5 buffer [126]. The process was optimized by using a design of experiments (DoE) approach to screen different internal diameters (ID) and the ratio between the two phases, which was found to influence the dimension of the segments and the residence time. Finally, the optimal conditions were then applied to the ADS-catalyzed conversion of FDP (**1**) into amorpha-4,11-diene (**2**) in 69% yield with 90 min of residence time.

**Scheme 5:**
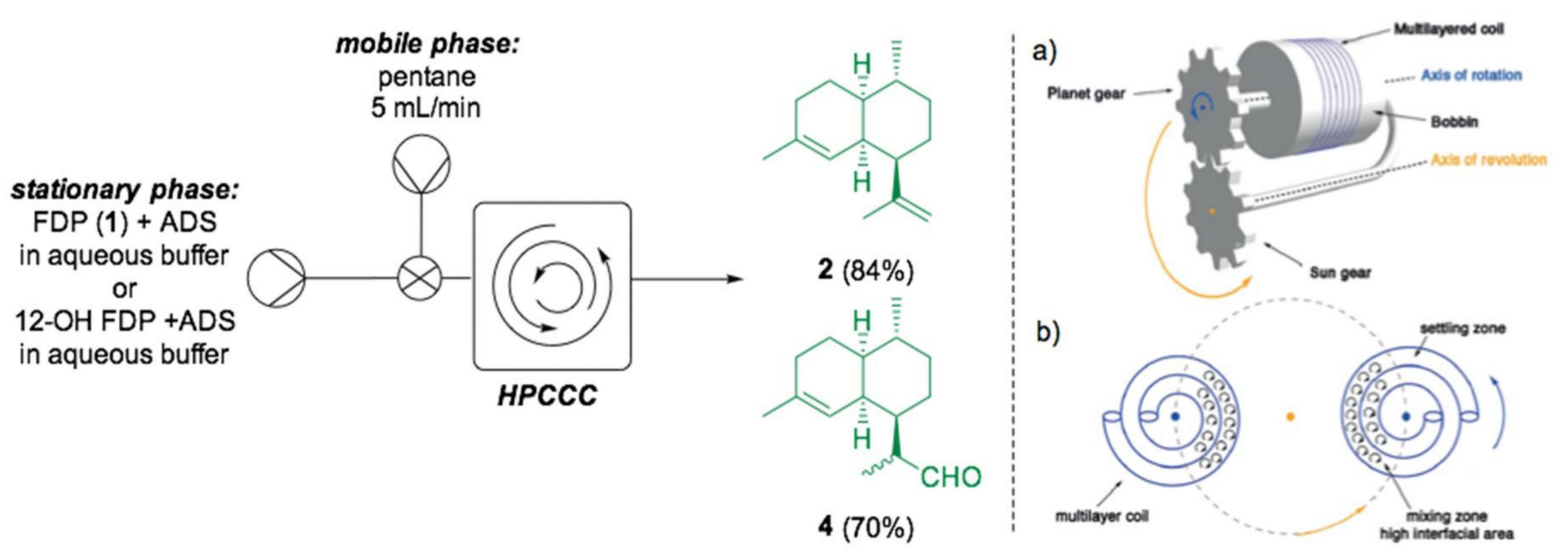
Amorpha-4,11-diene (**2**) and DHAA (**4**) biosynthesis in a HCCC system from FDP and 12-OH FDP, respectively. Schematic view of the HPCCC showing only one of the two bobbins for clarity (**a**) and of the mixing zone (**b**). Figure printed from reference [127].

The same system has been further accelerated by using extreme mixing under unusual reaction conditions. Recently, Huynh et al. reported the first example of high-performance countercurrent chromatography (HPCCC) applied to stereoselective phase transfer as well as biocatalytic reactions [127]. Liquid/liquid countercurrent chromatography (CCC) has been used in industry for the purification of complex mixtures of natural products as well as pharmaceuticals since 2015 [128], but it has not been considered a convenient method to reach extreme mixing until now. The basic concept behind CCC technology relies on the partition of solutes between two immiscible liquid phases, one of which is kept stationary through centrifugal forces. The system is composed of a series of two bobbins with a set of rotating gears (Scheme 5).

The stationary phase is pumped in the system first and then held stationary by spinning the bobbin where the PTFE coil is wrapped. Once the stationary state is reached, the mobile phase is pumped through the system [129]. The extreme mixing is made possible by the bobbin, which spins around its axis of rotation while rotating around its axis of revolution, creating more than two million mixing zones per hour [130]. For the synthesis of amorpha-4,11-diene (**2**), purified ADS and FDP (**1**) were loaded on the HPCCC analytical column (22 mL) as the stationary phase, while pentane was used as the mobile phase and pumped in a second moment. The optimal conditions were then applied to the synthesis of different sesquiterpenes, including amorpha-4,11-diene (**2**) and dihydroartemisinic aldehyde (DHAA, **4**), both artemisinin metabolic intermediates that have been reported to notably improve yields compared to both batch and segmented flow setups, with shorter residence times (up to 11 min).

**Scheme 6:**
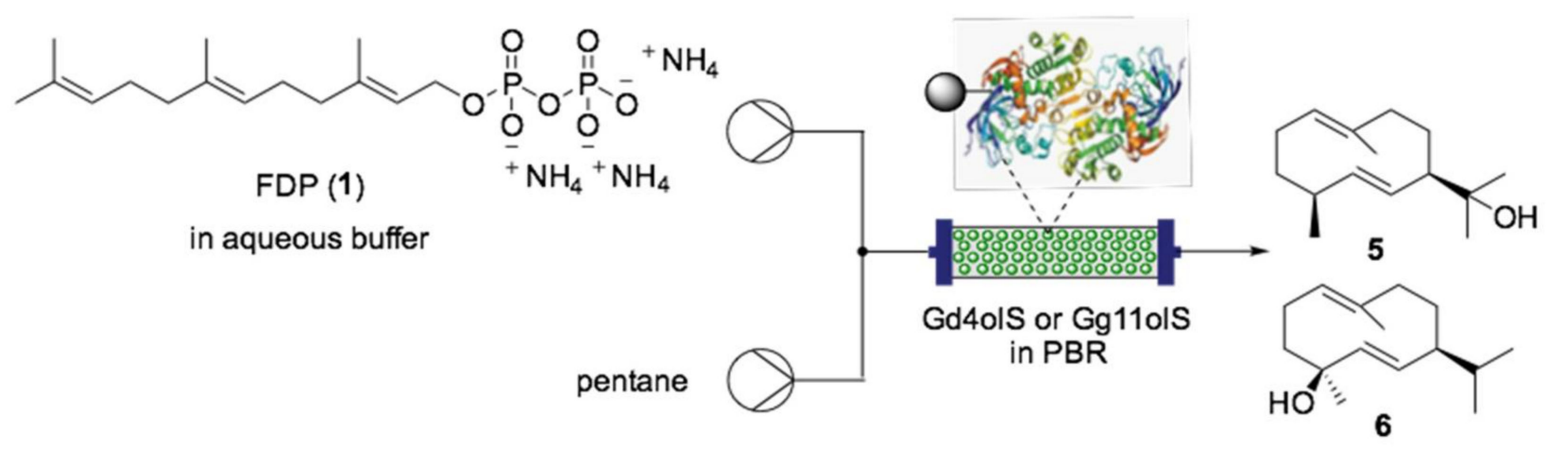
Continuous flow system for the production of sesquiterpenes **5** and **6** using immobilized enzyme; PBR = Packed-bed reactor [131].

In addition to slow product release, another aspect of biocatalytic synthesis of sesquiterpenes that can find benefits from flow chemistry is enzyme reusability and easier downstream processing obtained via enzyme immobilization [131]. In this regard, Valikhani et al. reported the performance of immobilized germacradien-4-ol synthase (Gd4olS) and germacradien-11-ol synthase (Gd11olS) in an organic-aqueous biphasic flow system for the synthesis of sesquiterpenes **5** and **6** (Scheme 6).

For this purpose, the authors used a highly porous glass carrier (EziG^TM^) coated with an organic polymer and a metal. The enzymes were immobilized by metal affinity for minimized conformational changes, then packed in an Omnifit^®^ glass column. The authors were able to prove an unaltered conversion for at least 50 cycles under continuous flow conditions. Intuitively, this method could also be applied to other enzymes, offering an attractive and easy scale-up method for industrial biosynthesis of valuable bioactive compounds such as amorpha-4,11-diene (**3**).

**Scheme 7:**
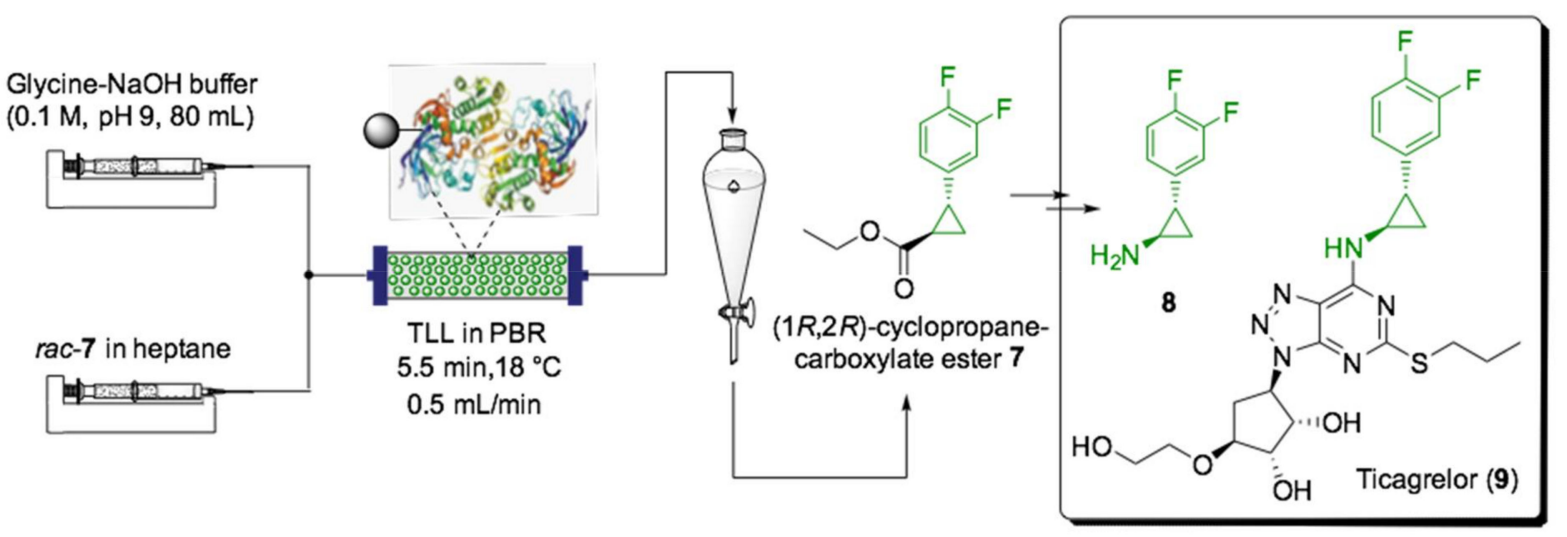
Schematic overview of flow setup for the synthesis of 1*R*,2*R*-**7**, a synthetic precursor for ticagrelor (**9**) [132].

While, to the best of our knowledge, no scale-up for the biosynthesis of pharmaceutically relevant terpenes has been reported yet, immobilized enzymes have already found several applications in the synthesis of different APIs and API precursors, even on a large scale. For instance, Turner et al. employed this approach for the synthesis of a ticagrelor building block [132]. Ticagrelor (**9**) is one of the world’s leading medicaments for the treatment of acute coronary symptoms, which presents (1*R*,2*S*)cyclopropylamine (**8**) as its main core (Scheme 7). The chiral amine **8** can be readily obtained starting from racemic cyclopropanecarboxylate ester (**7**), a low-value precursor that is easily accessible on a kilogram scale.

The enzymatic kinetic resolution was catalyzed by *Thermomyces lanuginosus* lipase (TLL), which was in turn immobilized covalently in Immobead 100 carrier (9820 units/g). The immobilized enzyme was then used in both batch and continuous flow systems in order to compare both biocatalytic systems. Under batch conditions, the immobilized lipase was contained in a “teabag” construction to avoid crumbling caused by stirring. In this case, the racemic ester **7** in heptane and glycine−NaOH buffer (0.1 M, pH 9.0) were subjected to hydrolysis at 18 °C for 23 h. Six reaction cycles were performed in batch mode, each one processing 2.21 mmol of **7** without any significant loss of enzyme activity or enantioselectivity. In flow mode, 10 mL solutions were pumped through the PBR containing immobilized TTL using two separate syringes, one containing glycine−NaOH aqueous buffer (1.1 M, pH 9) and another with *rac-***7** in heptane (0.2 M), set to 0.25 mL/min, with 40 min residence time. Notably, increased productivity was achieved under continuous-flow conditions as the space–time yield of the flow reactor was 64 times more efficient than the batch reactor (28.2 mmol·L^−1^·h^−1^ vs. 0.4 mmol·L^−1^·h^−1^). Moreover, a slightly higher selectivity for the target ester (1*R*,2*R*)-cyclopropane-carboxylate ester **7** in the flow system was achieved (*E* = 58 vs. 52).

**Scheme 8:**
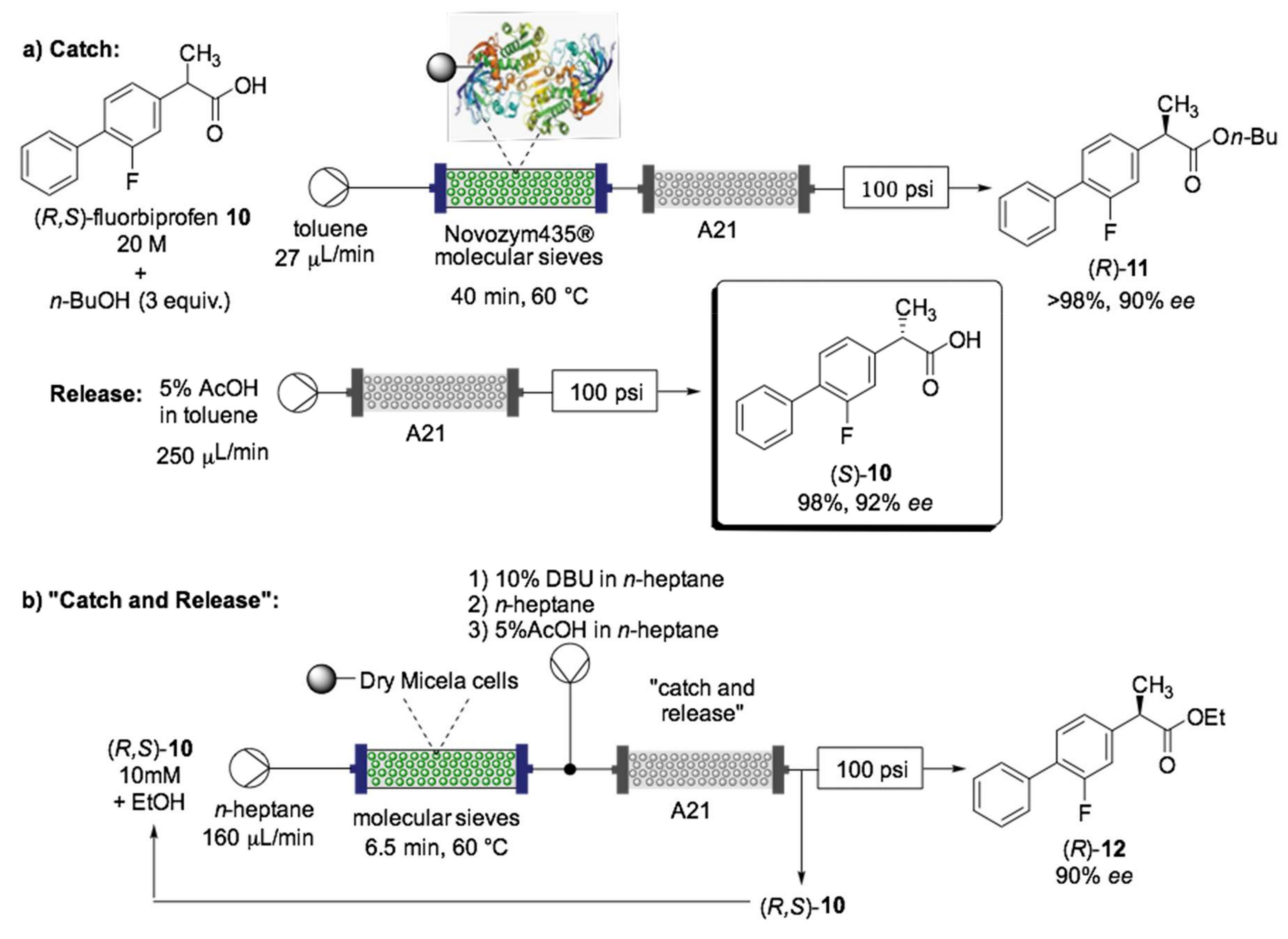
Schematic overview for the enzymatic kinetic resolution of flurbiprofen (**10**) under flow conditions, using immobilized lipase B from *Candida antarctica* (**a**) or immobilized-dry mycelia of *Aspergillus oryzae* (**b**) [134,135].

Enzymatic kinetic resolution is of great interest in the pharmaceutical industry because it allows an easy enantiomer separation, avoiding the main drawbacks of racemic drugs, such as side effects or low activity driven by a highly toxic, differently bioactive or simply inactive undesired enantiomer. For example, the kinetic resolution of racemic (*RS*)-flurbiprofen (**10**) is an attractive protocol since (*R*)-**10** is known for being a nonsteroidal anti-inflammatory drug (NSAID), while the counter enantiomer (*S*)-**10** has shown anticancer activity [133]. An efficient example of a flow bioreactor for the kinetic resolution of (*RS*)-flurbiprofen (**10**) was previously reported by Tamborini et al. in 2012 (Scheme 8) [134,135].

In one case, the kinetic resolution of *rac*-**10** was carried out by immobilized lipase B from *Candida antarctica* (Novozym 435^®^) in a packed-bed reactor (Scheme 8a) [134]. The temperature, residence time and stoichiometry ratio were screened in order to find the optimal conditions to obtain each enantiomer with a high degree of enantiomeric excess under flow conditions, and the conditions were then compared to the one in batch. In particular, a significant reduction of the reaction time (*r_flow_*) was registered (15–60 min vs. 6 h in batch mode), and the productivity was found to be 10 times higher in flow than in batch (8.40 vs. 0.84 µmol/min g). This system was further improved by adding a column containing a polymer-supported base (Amberlyst^®^ A21) for inline purification. This two-step “catch and release” protocol achieved pure acid (*S*)-**10** in 98% and 92% *ee,* respectively, avoiding tedious extraction/separation steps. A very similar flow bioreactor with immobilized-dry mycelia of *Aspergillus oryzae* was reported for the enantioselective esterification of *rac*-**10** into ethyl ester (*R*)-**12** (Scheme 8b) [135]. This case also obtained a reduction in *r_flow_* compared to batch as well as improved enantioselectivities.

**Scheme 9:**
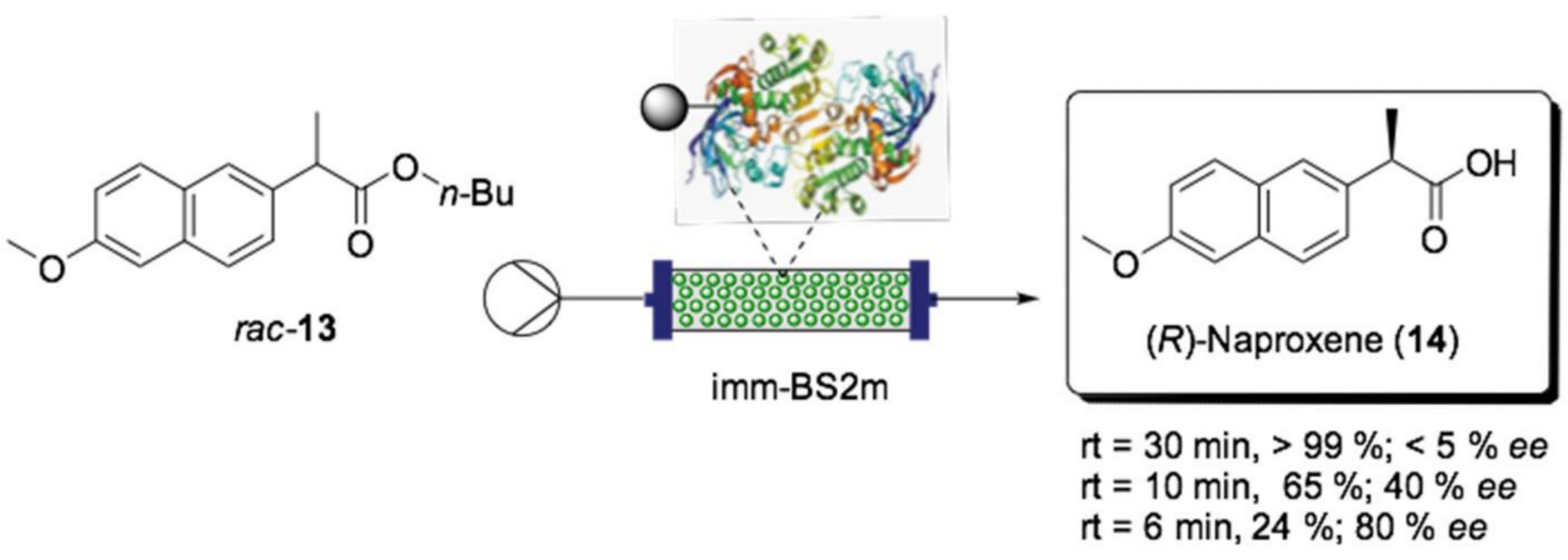
Schematic overview of the flow bioreactor for the enantioselective hydrolysis of naproxen ester **13** [136].

A continuation of this work was reported more recently by Contente and Paradisi et al., who studied the enantioselective cleavage of naproxen butyl ester (**13**, Scheme 9) into enantiomerically enriched acid using *Bacillus subtilis* esterase BS2m covalently bound on an agarose support [136]. After optimizing the reaction conditions, specifically in terms of naproxen ester solubility in phosphate buffer with the addition of 0.5% of the non-ionic surfactant Triton X-100, they were able to reach complete hydrolysis into (*R*)-**14** within 30 min of residence time. Interestingly, in batch, the same reaction took 48 h to reach 55% conversion. Finally, since BS2m showed selectivity for the (*R*)-enantiomer when investigated in the batch reactions, the residence time was shortened to check whether it could affect the facial selectivity and thus the degree of enantiopreference. With 10 min residence time, the conversion diminished to 65% with 40% *ee*, while at 6 min, the enantiomeric excess increased to 80%, and the conversion dropped to 24%.

**Scheme 10:**
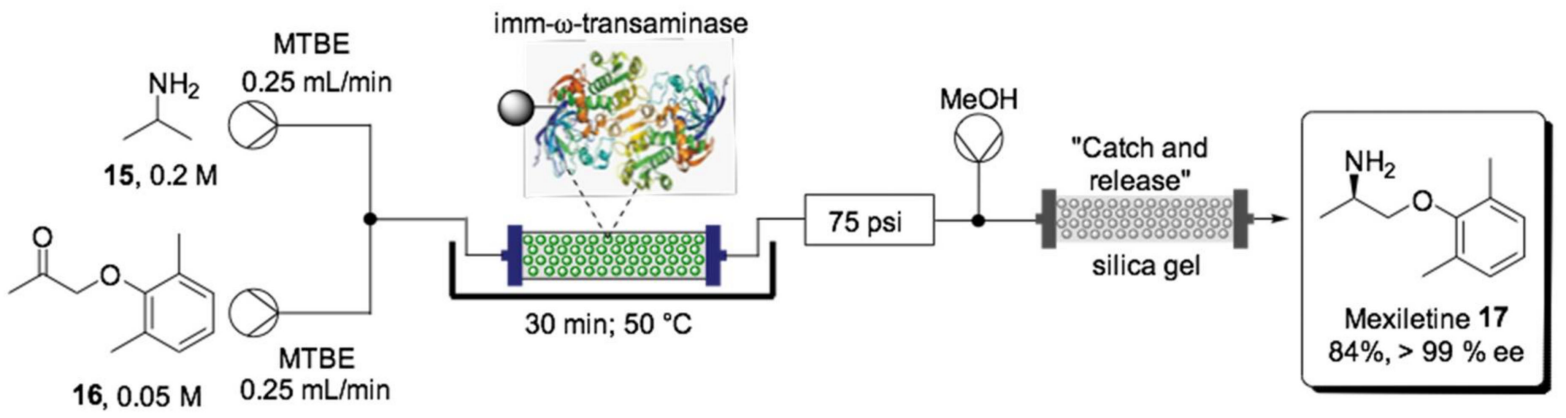
Schematic overview of the flow bioreactor for the enantioselective synthesis of mexiletine (**17**) [137].

Enzyme purification is well known to be a time-consuming and cost-inefficient step, which is why developing protocols that employ whole cells or lyophilized cells is an attractive alternative. This approach has the advantage of ensuring enzyme stability within its native medium and requires no external cofactor addition. An excellent example of flow biocatalysis for API synthesis is the continuous flow synthesis of mexiletine (**17,** Scheme 10) [137]. The antiarrhythmic, antimyotonic and analgesic oral drug mexiletine (**17**) was obtained by asymmetric amination of (2,6mimethylphenoxy)acetone (**16**) in the presence of isopropylamine (**15**) catalyzed by whole-cell *Escherichia coli* containing overexpressed (*R*)-selective *ω*-transaminase. The enzyme was immobilized in methacrylate polymer resin and held in a packed-bed reactor. A methyl *tert*-butyl ether (MTBE) solution containing ketone **16** and amine **15** was injected separately into the packed-bed reactor. Under optimized conditions, the transamination reaction yielded 84% of the enantiopure mexiletine (*ee* > 99%) at 50 °C within 30 min of residence time. It was noteworthy that the system operated continuously for 10 days, with only 10% loss of enzyme activity. Additionally, a practical silica gel cartridge was set up to catch the product and then release it by elution with a methanol downstream system, making the process more efficient and sustainable.

**Scheme 11:**
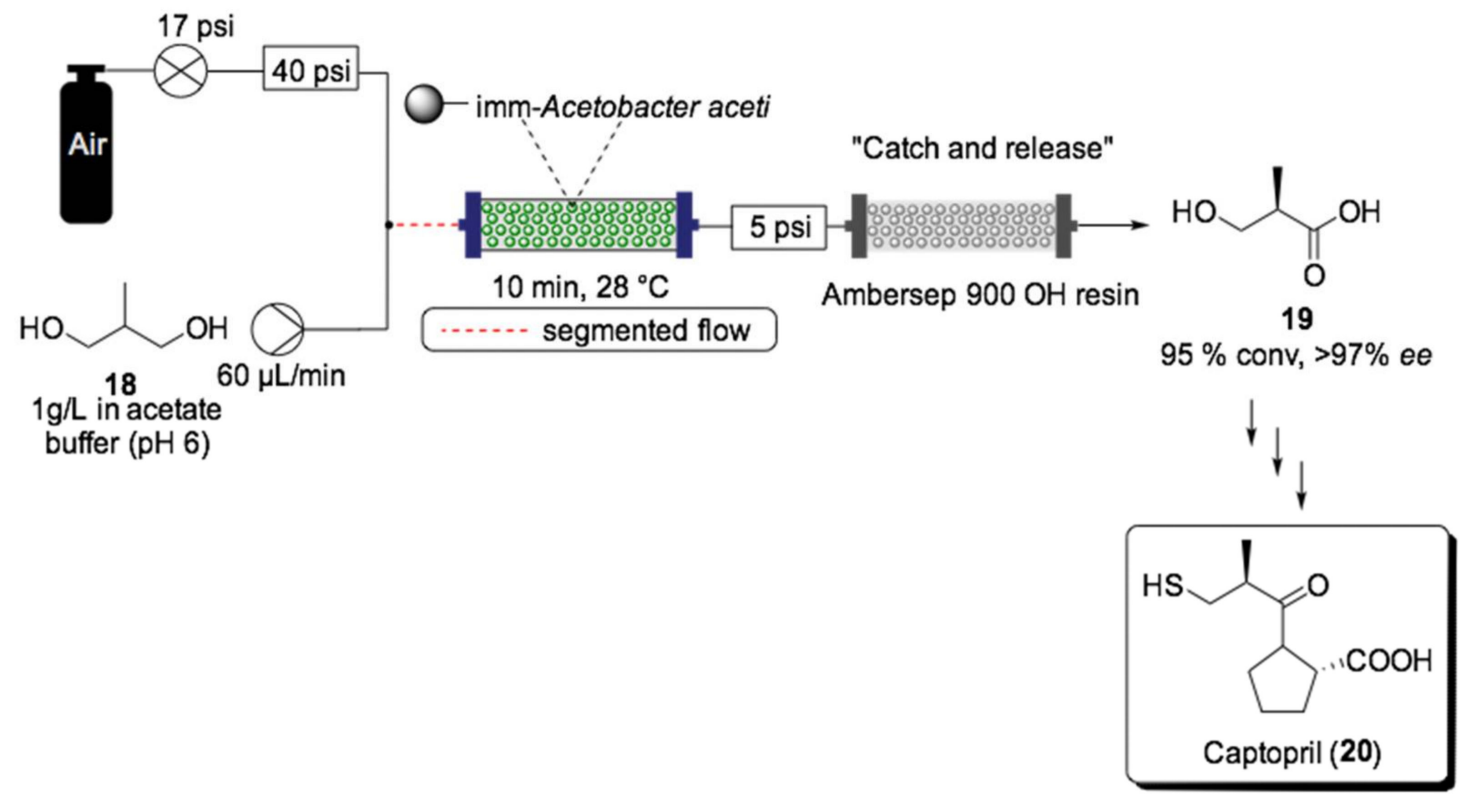
Schematic overview of the biocatalyzed heterogeneous oxidation of prochiral 2-methyl-1,3-propandiol (**18**) for the synthesis of captopril (**20**) in flow mode [138].

Another example of immobilized whole cells in flow was reported by Romano and Tamborini et al., who developed a four-step chemoenzymatic synthesis of captopril (**20**) using continuous flow systems (Scheme 11) [138]. Captopril is an angiotensin-converting enzyme (ACE) inhibitor that is widely prescribed as a drug for the treatment of hypertension. For this purpose, a PBR was prepared from whole cells of *Acetobacter aceti* MIM 2000/28 immobilized in alginate, which was used to catalyze the oxidation of one of the terminal alcohols in **18** to the carboxylic acid **19**, its first synthetic intermediate. This approach represents a great alternative to chemical oxidation, mitigating the risks associated with the exothermic oxidation reaction and offering a lower environmental impact by replacing the use of highly polluting reagents. The prochiral 2-methyl-1,3-propandiol (**18**) was dissolved in acetate buffer pH 6 and injected through the reactor after being mixed with oxygen through a segmented air–liquid flow stream. To ensure a constant flow, a 40 psi back pressure regulator (BPR) was applied before the air tank, and a 5 psi BPR was applied at the outflow of the reactor. This system showed stability under continuous operation for 10 h, yielding carboxylic acid with 95% maximum conversion (96–97% *ee*) with 10 min of residence time. For the downstream purification, a catch-and-released method was used to trap the acid. The retained acid on the basic resin (Ambersep 900 OH resin) was then released by the flow of 1 M hydrochloric acid and then lyophilized before moving to the next chemocatalytic steps. The three consecutive reactions, chlorination of carboxylic acid using thionyl chloride, coupling reaction with l-proline and the nucleophilic substitution of the chlorine with the thiol group, were also translated into a three-step continuous flow system, providing the desired API with 50% overall yield.

**Scheme 12:**
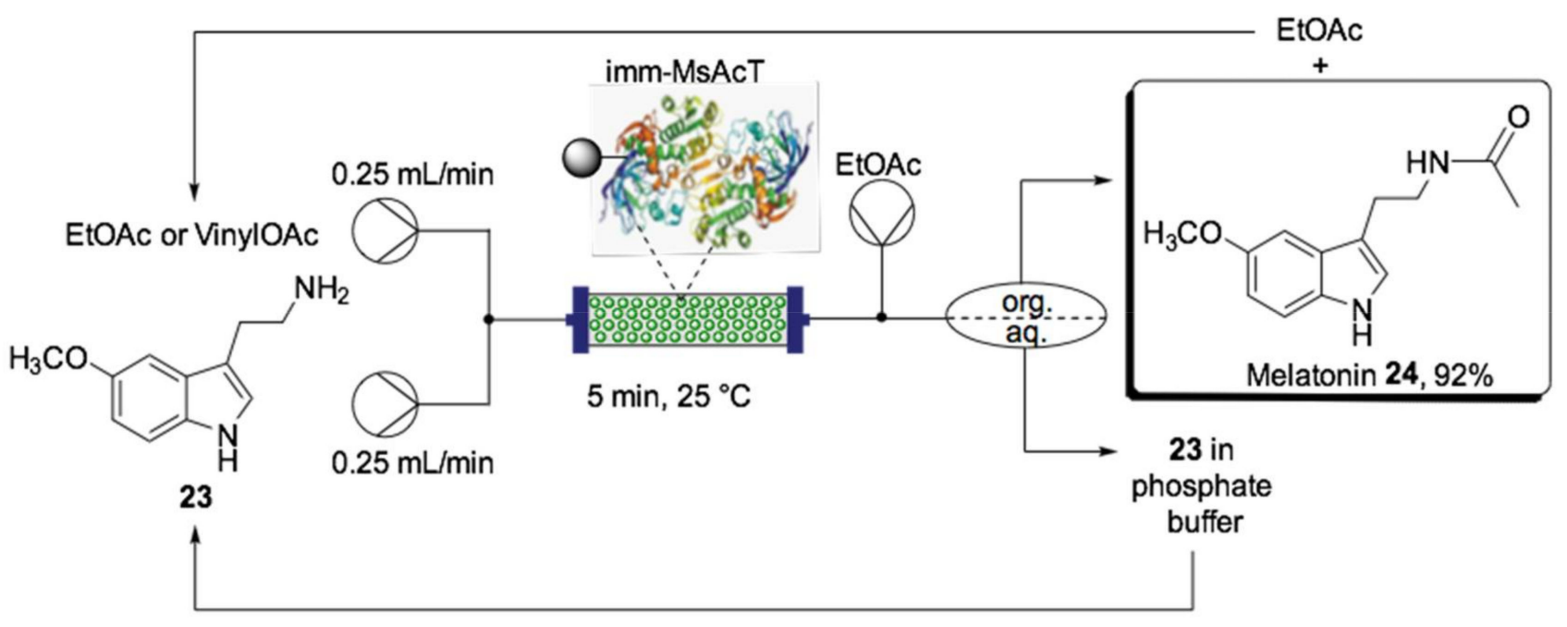
Schematic overview of the flow biocatalyzed synthesis of melatonin (**24**) using a closed-loop system [139].

Although the “catch-and-release” strategy was proven to improve the overall sustainability of the system, the interruption of the workflow to recover the trapped product could be a limitation. To overcome this, liquid–liquid extraction was adopted when the solubility of the product in an organic solvent was suitable to allow its separation in a biphasic system [139]. In 2019, Contente et al. reported the biocatalytic synthesis of melatonin (**24**) and analogs via *N*-acetylation of tryptamines catalyzed by *Mycobacterium smegmatis* transferase (MsAcT) in a continuous flow process. This system used a Zaiput liquid–liquid separator to purify the product, which allowed the recovery of the unreacted reagent and its recirculation back in the flow system by a closed-loop, then the product was obtained from the organic phase by evaporation (Scheme 12). The biocatalytic process using this flow reactor technology was a good option for *N*-acetylation because the acetylation reaction was more efficient than hydrolysis due to the constant product removal, making it a cleaner, more efficient and sustainable protocol. A solution of amine (**23**) in phosphate buffer (0.1 M, pH 8.0) and acetyl donor (ethyl acetate or vinyl acetate) was pumped separately into the continuous flow system, forming a segmented liquid–liquid phase that flowed through the packed-bed column containing MsAcT immobilized on glyoxyl agarose beads. With the reaction parameters of 5 min residence time and 0.5 M vinyl acetate **23** at 25 °C, as well as atmospheric pressure, 96% conversion and 92% isolated yield for **24** was obtained. It was also noteworthy that, when vinyl acetate was used for continuous flow production of melatonin over 24 h, a space–time yield of 36.9 g·day^−1^ with 99 mmol·mg^−1^ of catalyst productivity was achieved.

**Scheme 13:**
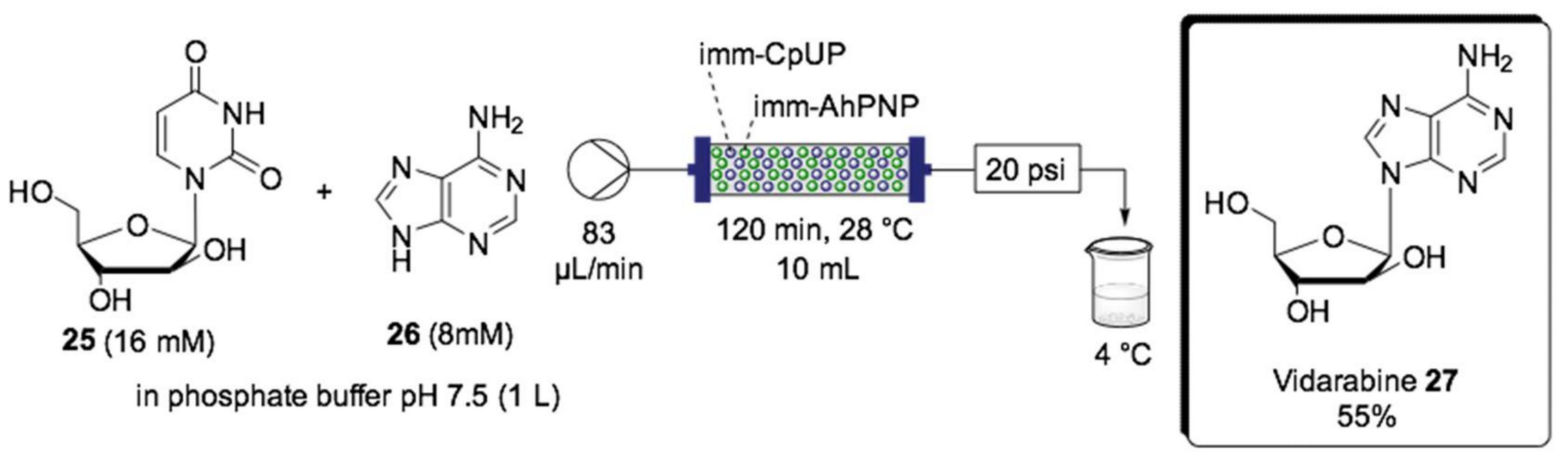
Schematic overview for the flow biocatalyzed synthesis of antiviral vidarabine (**27**) [140].

The preparation of some compounds can sometimes be simplified by using biocatalysts, which reduce the number of steps and avoid byproduct formation. Nucleoside phosphorylases are a good example of this approach, which have paved the way for nucleoside synthesis. A notable example is the synthesis of the nucleoside of pharmaceutical interest vidarabine (**27**), reported by Tamborini et al. [140]. The nucleoside phosphorylases catalyzed the transfer of the sugar residue **25** to a second nucleobase, leading to the production of a new nucleoside upon transglycosylation reaction. The coupling of two nucleoside phosphorylases is often necessary when transglycosylation occurs between a purine and a pyrimidine base [140]. Thereby, for the synthesis of vidarabine (**27**), a bioreactor containing co-immobilized uridine phosphorylase from *Clostridium perfringens* (CpUP) and a purine nucleoside phosphorylase from *Aeromonas hydrophila* (AhPNP) was used (Scheme 13). The bioreactor was prepared by in-flow coimmobilization of CpUP and AhPNP on glyoxyl agarose and EziG^TM^. The operational stability for the EziG^TM^ bioreactor was lower than that of the glyoxyl-agarose bioreactor. After 24 h of operation, an energy efficiency reduction of 10% was reported due to leaching of the enzyme. The continuous-flow synthesis of **27** was carried out at gram scale starting from 1 L of stock solution containing arabinofuranosyl uracil (araU, **25**) as the sugar donor and adenine as the sugar acceptor (**26**), which flowed through the reactor with a residence time of 120 min at 28 °C. The low solubility of vidarabine (araA, **27**) allowed it to be collected by precipitation in a cold vessel (4 °C) outside the reactor upon filtration. The operational stability was proven by 8 days of continuous work, which supplied a 55% yield of **27** in high purity (>99%).

This process was further investigated by Rinaldi et al., who also compared the performances of a monoenzymatic vs. a bienzymatic system using immobilized enzyme reactors (IMERs) for the synthesis of other pharmaceutically interesting nucleoside analogs [141]. In this case, the coimmobilization of CpUP and AhPNP was investigated using an aminopropyl silica monolithic support. The CpUP/AhPNP-IMER was placed in a chromatographic system, and the reaction mixture containing **25** (araU) as the sugar donor and **26** as the sugar acceptor was continuously pumped at 0.5 mL/min through the bioreactor at 37 °C to achieve 60% conversion into the desired vidarabine **27** within 24 h of recirculation in CpUP/AhPNP-IMER.

**Scheme 14:**
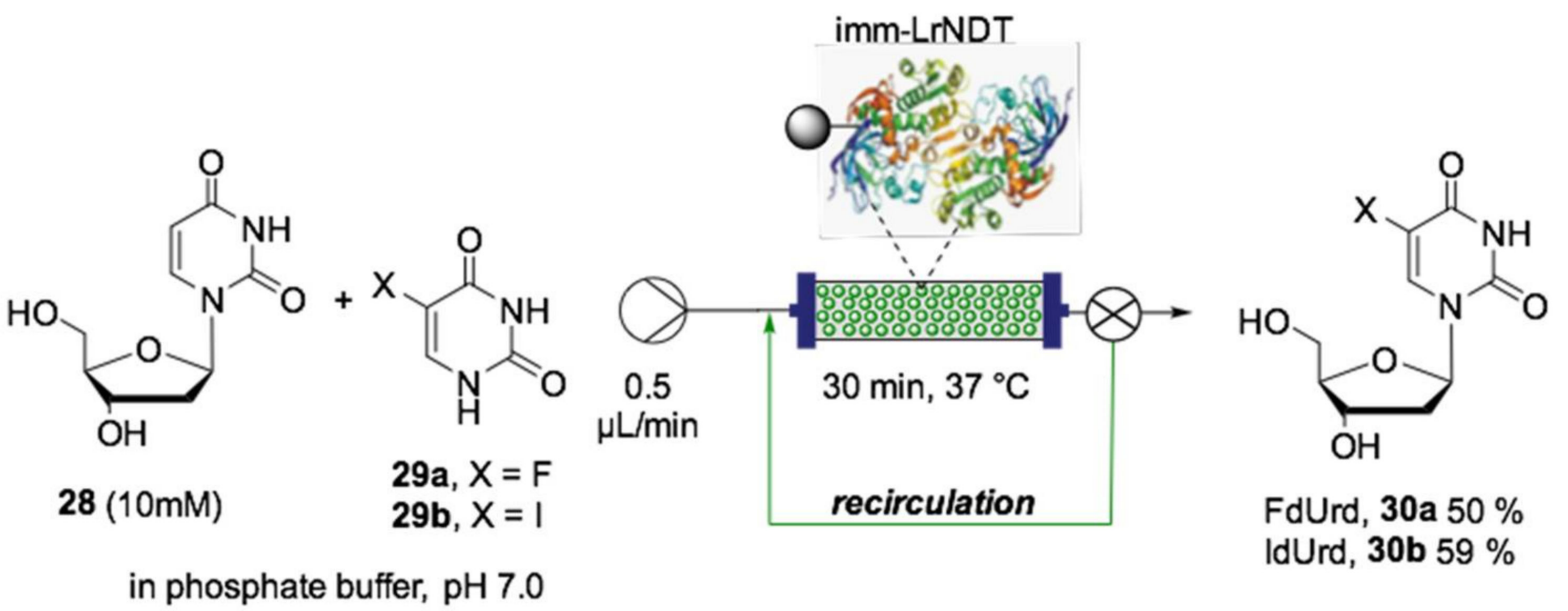
Schematic overview of the flow biocatalyzed synthesis of pharmaceutically interesting nucleosides [141].

Additionally, two pharmaceutically interesting nucleosides, including the antineoplastic agent 5-fluoro-2′-deoxyuridine (FdUrd, **30a**) and antiherpes drug 5-iodo-2′-deoxyuridine (IdUrd, **30b**) were synthesized using a monoenzymatic reactor containing nucleoside 2′-deoxyribosyltransferase from *Lactobacillus reuteri* (LrNDT) immobilized on a monolithic epoxy silica column. The biotransformation was performed using 2′-deoxyuridine (dUrd, **28**) as the sugar donor and nucleosides (**29**) as the sugar acceptor, affording the desired nucleosides **30a** and **30b** at 50% and 59% conversion, respectively, within 30 min of residence time (Scheme 14). When the CpUP/AhPNP-IMER was used on **28** and **29**, **30a**–**b** were achieved with only 37% and 26% conversion, respectively, after 2 h of recirculation, proving the monoenzymatic system to be more efficient for this type of biotransformation.

**Scheme 15:**
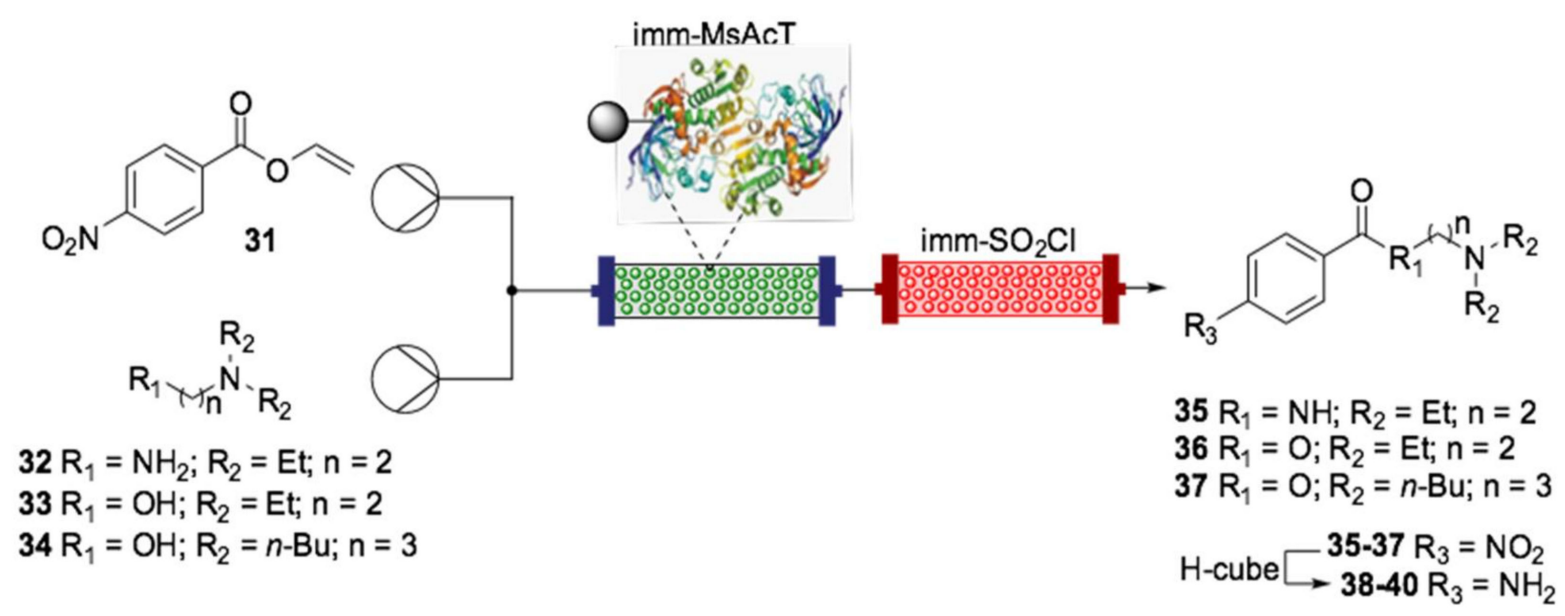
Schematic overview of the flow synthesis of procainamide (**38**), procaine (**39**) and butacaine (**40**) using immobilized MsAcT enzyme [142].

A chemoenzymatic approach for the synthesis of the blockbuster drugs procainamide (**38**), procaine (**39**) and butacaine (**40**) was carried out very recently by Tamborini et al. (Scheme 15) [142].

The merger of biocatalysis and flow chemistry was proven to be powerful by using the acyltransferase enzyme from *Mycobacterium smegmatis* (MsAcT) immobilized onto glyoxyl agarose, which enabled an easy incorporation to flow reactors, a controlled fluid dynamic and, as a result, excellent process efficiency. Vinyl 4-nitro-benzoate (**31**), used as the acyl donor, and compounds **32**, **33** and **34**, used as the nucleophilic counterparts, were solubilized in toluene, mixed in a T-piece and pumped into the bioreactor where the reaction took place, which was kept at 28 °C. Quantitative yield was obtained within 7 min of residence time (rt) using amine **34**. Interestingly, the reaction rate reached 28 µmol min^−1^ mg_enzyme_^−1^, more than twice that obtained in the batch, which required 24 h to be completed. Being alcohols that were less nucleophilic than the primary amine, a slight modification of the optimized protocol was necessary for the synthesis of the ester derivatives (compounds **36** and **37**). The inline purification by means of immobilized sulphonyl chloride connected with the bioreactor was capable of efficiently trapping the excess of the nucleophile, permitted compounds **35**–**37** to be obtained in their pure form. Finally, the target APIs (compounds **38**–**40**) were prepared by the hydrogenation reaction carried out in an H-Cube using a 10% Pd/C cartridge.

### 4.2. Flow Biocatalysis Applied to Multi-Step Synthesis

Although the advantages of single-step transformations in flow systems have been proven, the real power of continuous flow is in multi-step systems, which could provide interesting pharmaceutical applications [143]. In these systems, several reactors are connected in series with a unique flowing stream in which intermediates are synthesized in situ and used immediately for the following steps without purification or after inline purification. Furthermore, in biocatalysis, multi-step systems are extremely valuable because they allow chemoenzymatic transformations to be performed that would otherwise be impossible in one pot due to the incompatibility of reaction conditions.

**Scheme 16:**
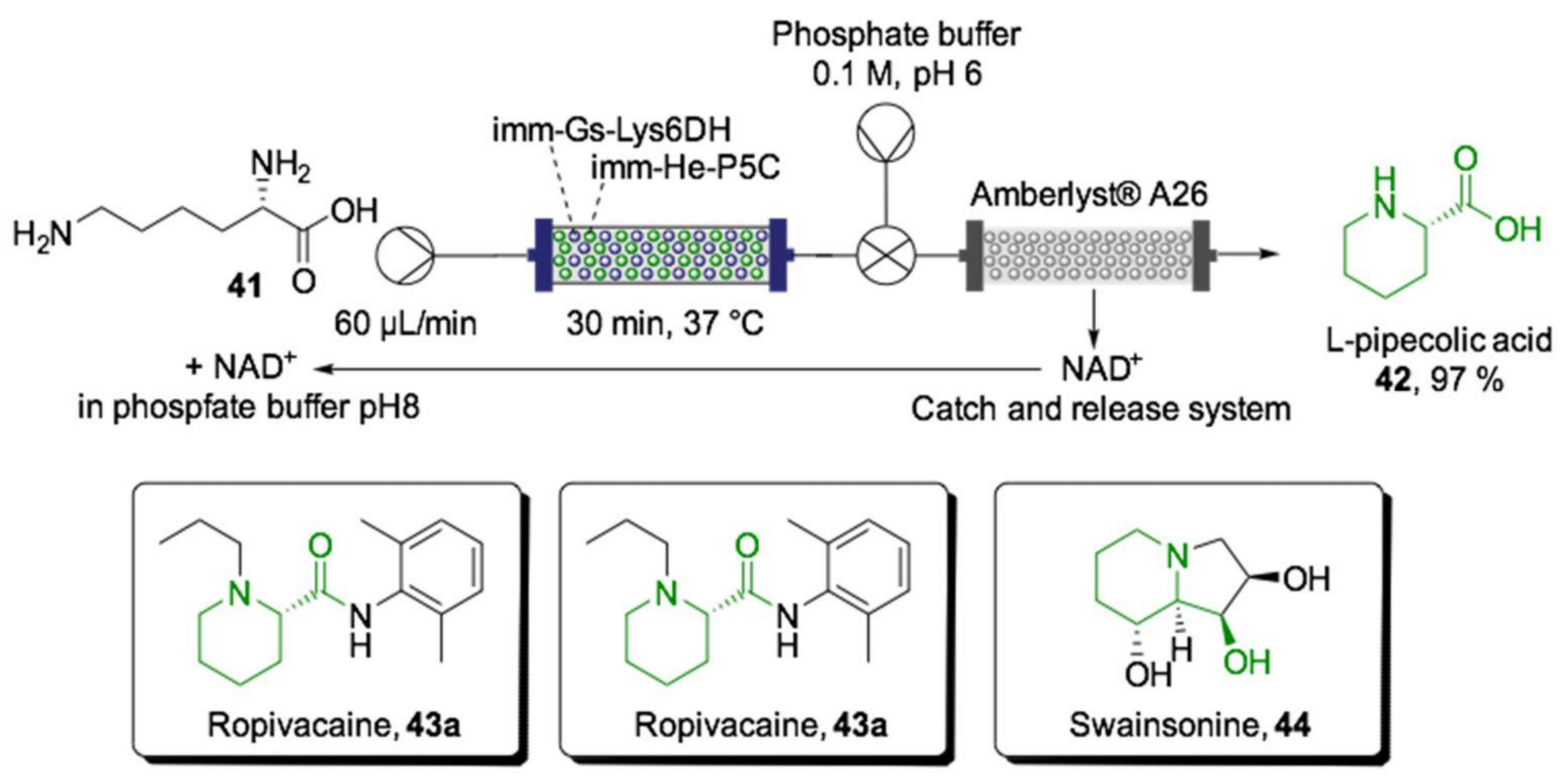
Schematic overview of the cascade flow biocatalyzed synthesis of l-pipecolic acid **42** using coimmobilized enzymes in a PBR [144].

The “catch-and-release” strategy for downstream purification is also very useful for recovering and recycling precious cofactors. The use of cofactor-dependent enzymes on large scale biocatalysis is still limited since costly cofactors are lost in downstream processing. This issue was addressed in the synthesis of l-pipecolic acid (**42**), a structural core present in several APIs, such as anesthetic ropivacaine (**43a**) and mepivacaine (**43b**), antitumor swainsonine (**44**) or antibiotics [144]. For preparation of the l-pipecolic acid using flow biocatalysis, a cascade reaction for l-lysine (**41**) deamination and reduction of cyclic imine was accomplished (Scheme 16). A NAD-dependent lysine dehydrogenase from *Geobacillus stearothermophilus* (Gs-Lys6DH) and a pyrroline-5-carboxylate reductase (P5C) from the *Halomonas elongata* were immobilized in agarose microbeads and packed into an Omnifit^®^ glass column (PBR volume: 1.6–2 mL). Interestingly, comparing the batch conditions, the turnover number of each free enzyme was 0.04 μmol·min^−1^·mg^−1^, while under optimized flow conditions, it increased to 0.33 μmol·min^−1^·mg^−1^, which was almost a 10-fold increase. The bienzymatic system (2–2.5 g) was fed with a solution containing l-lysine (21, 10 mM) and NAD^+^ (1 mM) in pH 8.0 potassium phosphate buffer (50 mM) pumped at 0.1 mL·min^−1^.

The l-pipecolic acid was obtained in >99% molar conversion with 30 min of residence time. The system was coupled to a scavenger column packed with Amberlyst^®^ A26 to catch the cofactor and let the product flow out of the reactor. The trapped cofactor was easily recovered (87% yield) by flushing a slightly acidic buffered solution (pH 6) through the scavenger column. The resin was then regenerated with 1 M NaOH for 10 min and reused in a new process. The complete flow system worked continuously over 180 min to afford 97% of the desired l-pipecolic acid **42**.

**Scheme 17:**
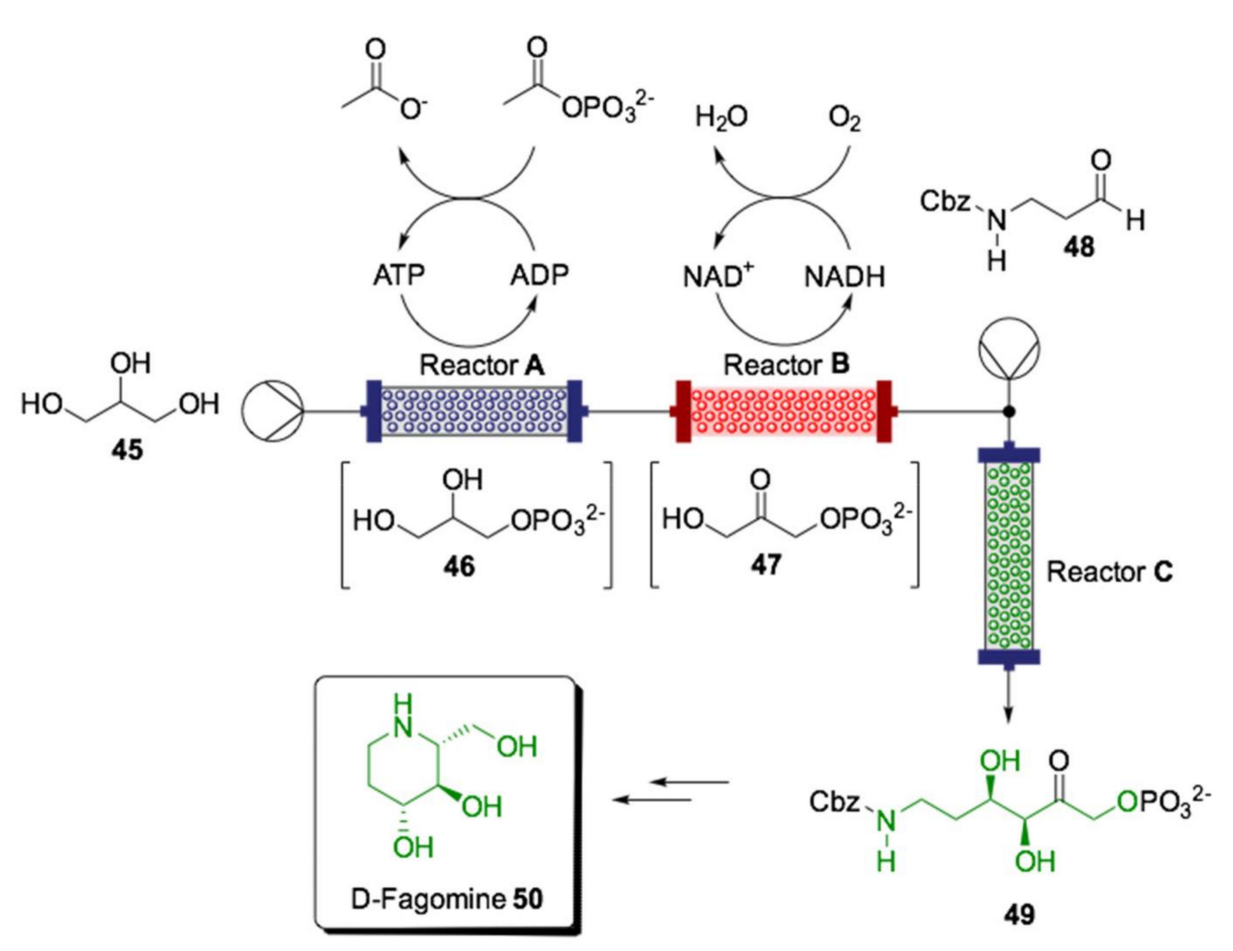
Schematic overview for the synthesis of antidiabetic drug d-fagomine (**50**) using engineered enzymes that retain and regenerate their cofactors in a multistep flow bioreactor [145].

While flow chemistry has proven to be an excellent technology to resolve several issues found in biocatalytic reactions, the use of pure cofactor-dependent enzymes in continuous flow systems is still limited, in particular for multistep processes, mainly due to cofactor recycling, which requires a “washing” step for freeing the cofactor from the scavenger column as well as providing a low total turnover number. For this reason, cofactor immobilization and site-specific immobilization systems have been developed by Scott et al. to overcome these barriers through a sophisticated protein engineering strategy [145]. Cofactor-dependent enzymes were combined in an assembly that retained and recycled their cofactors to synthesize an advanced intermediate in the production of an antidiabetic drug (d-fagomine, **50**) into a single continuous operation (Scheme 17). Three bioreactors were coupled in series to perform phosphotransfer, oxidation and, finally, carboligation (Reactor C), each one containing a specific biocatalytic system. The most significant innovation of this system was the modular design of the biocatalysts, composed of an enzyme, a cofactor recycling system and a conjugation domain, to link the biocatalyst to the support. Moreover, all modules were produced in a single molecule with short amino acid spacer regions by recombinant DNA technology. The enzymes used for the phosphor-transfer biotransformation in Reactor A were a *Thermococcus kodakarensis* glycerol kinase (GlpKTk) and a *Mycobacterium smegmatis* acetate kinase (AceKMs). For the second packed-bed reactor (Reactor B), *E. coli* glycerol-3-phosphate dehydrogenase (G3PD Ec) and the water-forming NADH oxidase from *Clostridium aminovalericum* (NOXCa) were used. Finally, the carboligation reactor was responsible for a cofactor-independent aldolase-catalyzed aldol addition (Reactor C). For this step, a fructose aldolase (FruA) homologue from *Staphylococcus Carnosus* was selected. The system was initially fed by pumping glycerol (**45**), which underwent phosphorylation in the first reactor. Glycerol-3-phosphate (**46**) was then oxidized to dihydroxyacetone phosphate (DHAP, **47**) in the second reactor. DHAP was mixed with NCbz-3-aminopropanal (**48**) before passing into the third reactor for stereoselective aldol addition generating **49**. These carefully designed and complete multienzyme assemblies demonstrate that investment in new technologies is reflected in high productivity, with up to 70 g L^−1^ h^−1^ g^−1^ space–time yields for **50**, >99% *ee* and ~17,000 total turnover numbers in the ATP-dependent phosphorylation reactor. The stability is also evidenced by the continuous flow operation of three cycles of 8 h at 23 °C with storage at 4 °C between runs without exogenous addition of cofactors.

**Scheme 18:**
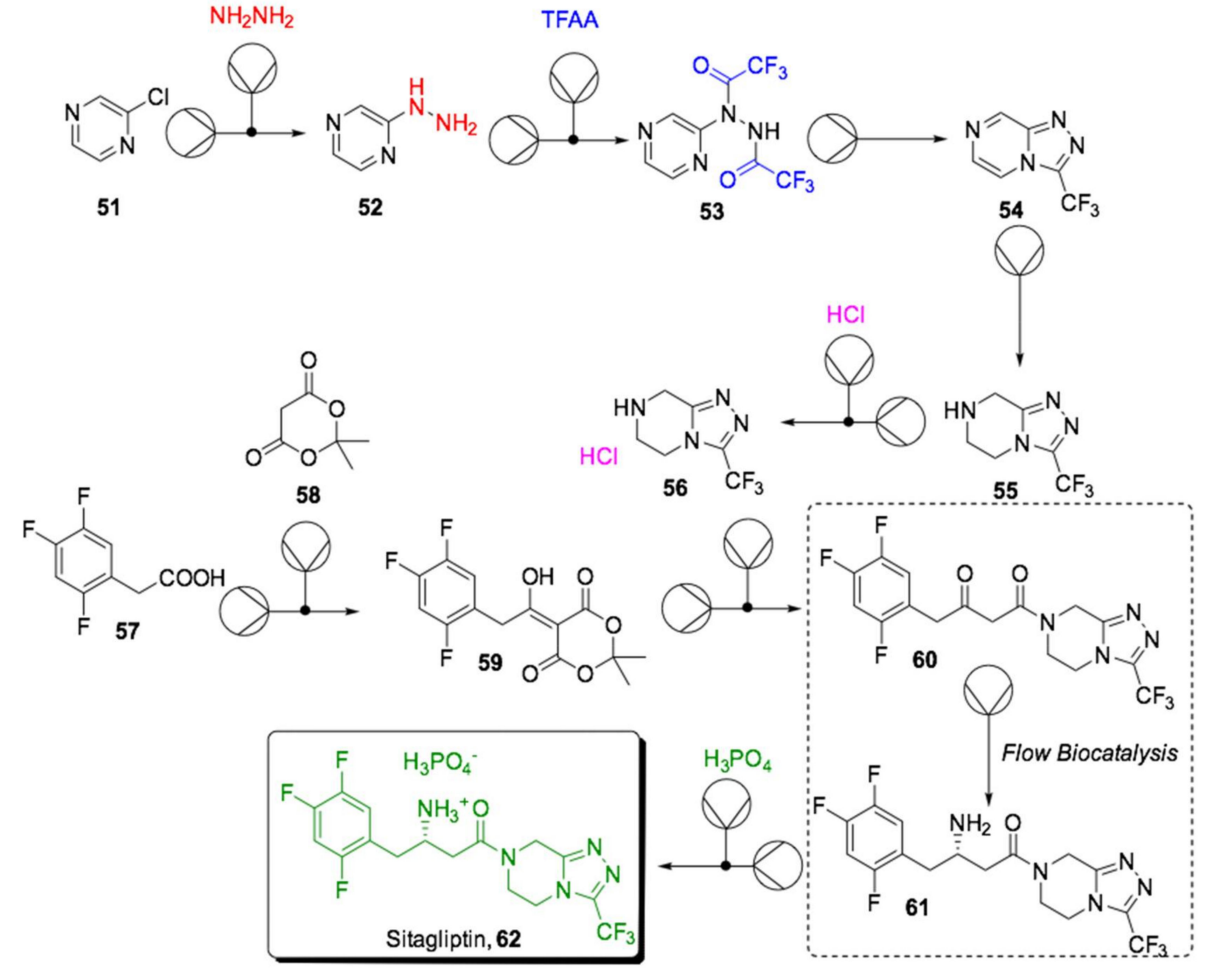
Simplified flowsheet for the biocatalytic continuous manufacturing of antidiabetic drug sitagliptin (**62**); TFAA = Trifluoroacetic anhydride [147].

As previously mentioned, the intrinsic potential of continuous flow biocatalysis is to ensure quality, reproducibility and efficiency while reducing waste and costs compared to a similar process in batch. Therefore, developing a multistep continuous manufacturing process that includes biocatalytic steps will lead to highly productive and cost-efficient industrial plants with a lighter environmental footprint as well as meeting the principle of quality by design (QbD), which are all highly desirable aspects that pharmaceutical companies are seeking. While it is true that some biotransformations have been translated into “continuous-mode” pilot-plants by using continuously-stirred tank reactors (CSTRs) in series [146], the application of plug-flow microreactor technology for the scale-up of multistep API synthesis is currently understudied. An initial example was recently reported by Ho et al., who designed an end-to-end continuous flow system that included a biocatalyzed step for the synthesis of the antidiabetic drug sitagliptin (**62**, Scheme 18) [147].

Traditionally, the conversion of ketone **60** into chiral *β*-amino amide (**61**) is performed using a chiral rhodium catalyst, which is not an ideal synthetic strategy when designing an industrial plant. Sitagliptin (**62**) is the most commonly used antidiabetic drug along with insulin injections; therefore, a more sustainable production pathway would be desirable. In 2010, Savile et al. reported the use of novel transaminase for a highly selective (>99.5% *ee*), efficient and greener biotransformation of **60** into **61** compared to the traditional chemocatalytic method [48]. Ho and coworkers translated the biotransformation reported by Savile et al. into plug-flow microreactors whose small dimension provided an improved mass and heat transfer that was incorporated into the newly designed multistep continuous flow system. The latter consisted of several mixing units, reactors, liquid−liquid extractors for downstream separation and inline crystallizers, as well as online analytical devices, and was applied to a more sustainable production of API **62** starting from chloropyrazine (**51**). The process was run in continuous-mode, which included a main stream, and several recycling and waste streams. Several evaporator units were included in order to evaporate and recycle the organic solvent, reducing the environmental impact. For the main stereoselective biotransformation in flow, a free-enzyme (transaminase) strategy was chosen to avoid leaching of the immobilized enzyme over time and to provide an easier enzyme recycling stream. Furthermore, an online analytical optimization for productivity was performed using a surrogate-based optimization (pySOT) programmed in Python, which facilitated the identification of the global optimum within a short time. The optimal productivity for the steady state was found to be 2.6 · 10^−2^ mol h^−1^, which could easily be scaled up to meet the industrial demand by “numbering-up” multiple parallel microreactors. Finally, the environmental footprint for a potential industrial process was carefully analyzed. The E-factor is one of the most commonly used green chemistry parameters, and it is defined as the ratio between the amount of generated waste over the amount of API. It was computed to be significantly lower than the E-factor of an industrial batch process (53 vs. 200), confirming the environmental benefits attributed to flow biocatalysis.

## 5. Flow Biocatalysis Applied to the Synthesis of Natural Products and Aromas

Besides the above-reported synthesis of terpenes having a direct role in API preparations, some interesting examples of flow biocatalysis applied to the synthesis of natural products were recently reported. They are worth mentioning because of their importance in the pharmaceutical and nutraceutical industry as fragrances, aromas or food additives.

**Scheme 19:**
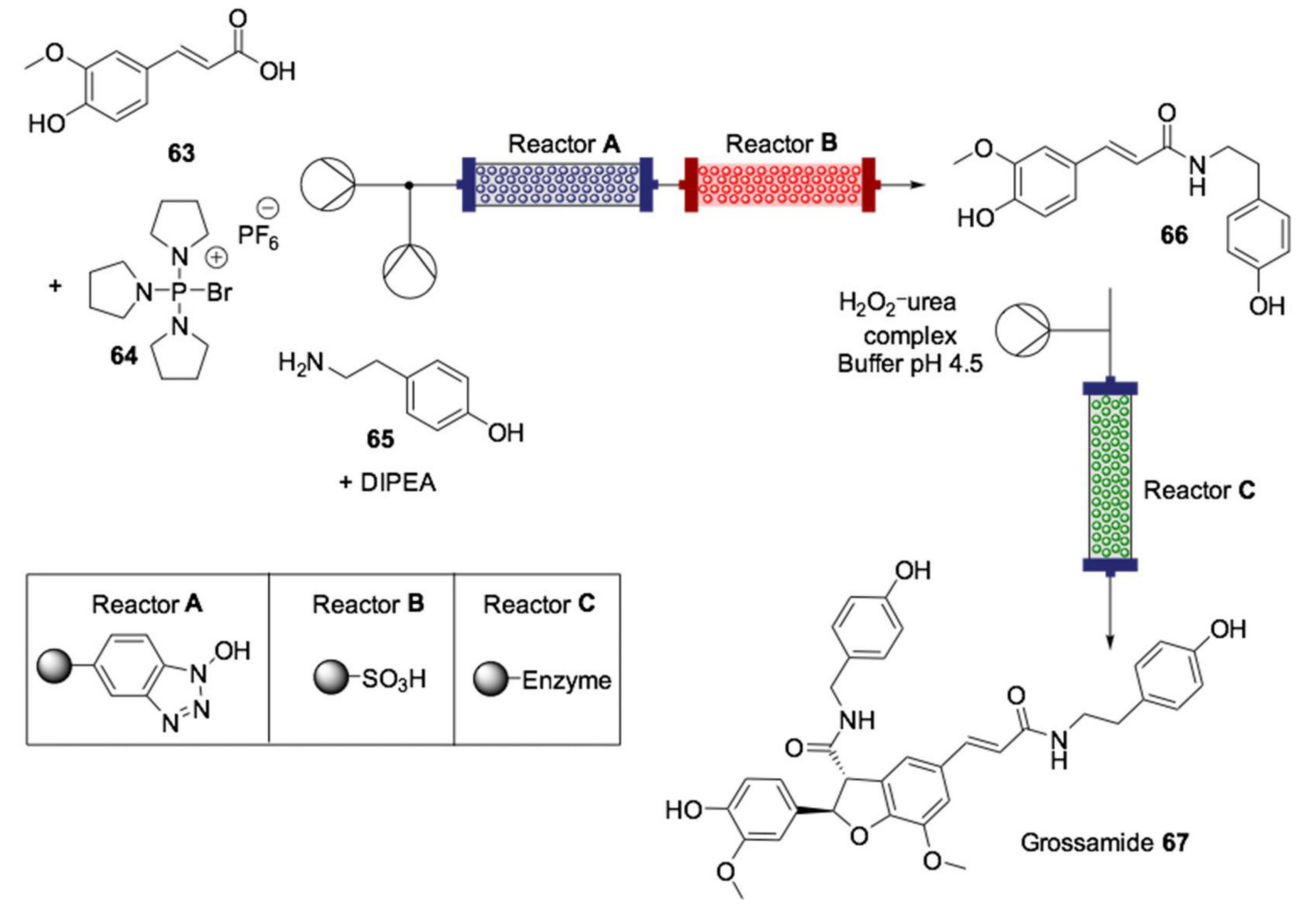
Schematic overview of the multistep flow synthesis of grossamide (**67**) [151].

The synthesis of natural products, perfumes and fragrances is one of the areas of chemistry experiencing significant expansion since there is a growing global demand for these raw materials [148]. Although most of these compounds can be extracted from natural sources, these extraction procedures are, in most cases, not environmentally adequate due to the large amount of plants required for industrial-scale production. In addition, the high cost and the work-intensive process of performing the extraction procedures make the development of synthetic methodologies a challenging alternative to overcome these issues [149]. In this context, biocatalysis associated with flow chemistry has emerged as an efficient and sustainable strategy for obtaining these compounds, and many procedures have been reported that demonstrate a concrete applicability. Grossamide **67** is a natural product that can be isolated from several fruits and seeds. It belongs to the class of lignan amides, which are promising molecules from a biological and agrochemical point of view [150]. The total synthesis of this neolignan was described by Ley’s group using a fully automated continuous-flow reactor containing immobilized reagents packed in columns [151]. The methodology consisted of the coupling of ferulic acid (**63**) with tyramine (**65**), followed by dimerization and intramolecular cyclization (Scheme 19).

The last step of the synthesis was characterized by the passage of the flow containing the building block **66** through a column loaded with urea-hydrogen peroxide and sodium dihydrogen phosphate buffer (pH 4.5) followed by another column packed with the immobilized enzyme horseradish peroxidase (type II) on silica. Interestingly, the developed protocol was scalable and demonstrated the efficiency of the use of flow biocatalysis in the preparation of 2-aryl-2,3-dihydro-3-benzofurancarboxyamide neolignan grossamide (**67**) in a flow system.

**Scheme 20:**
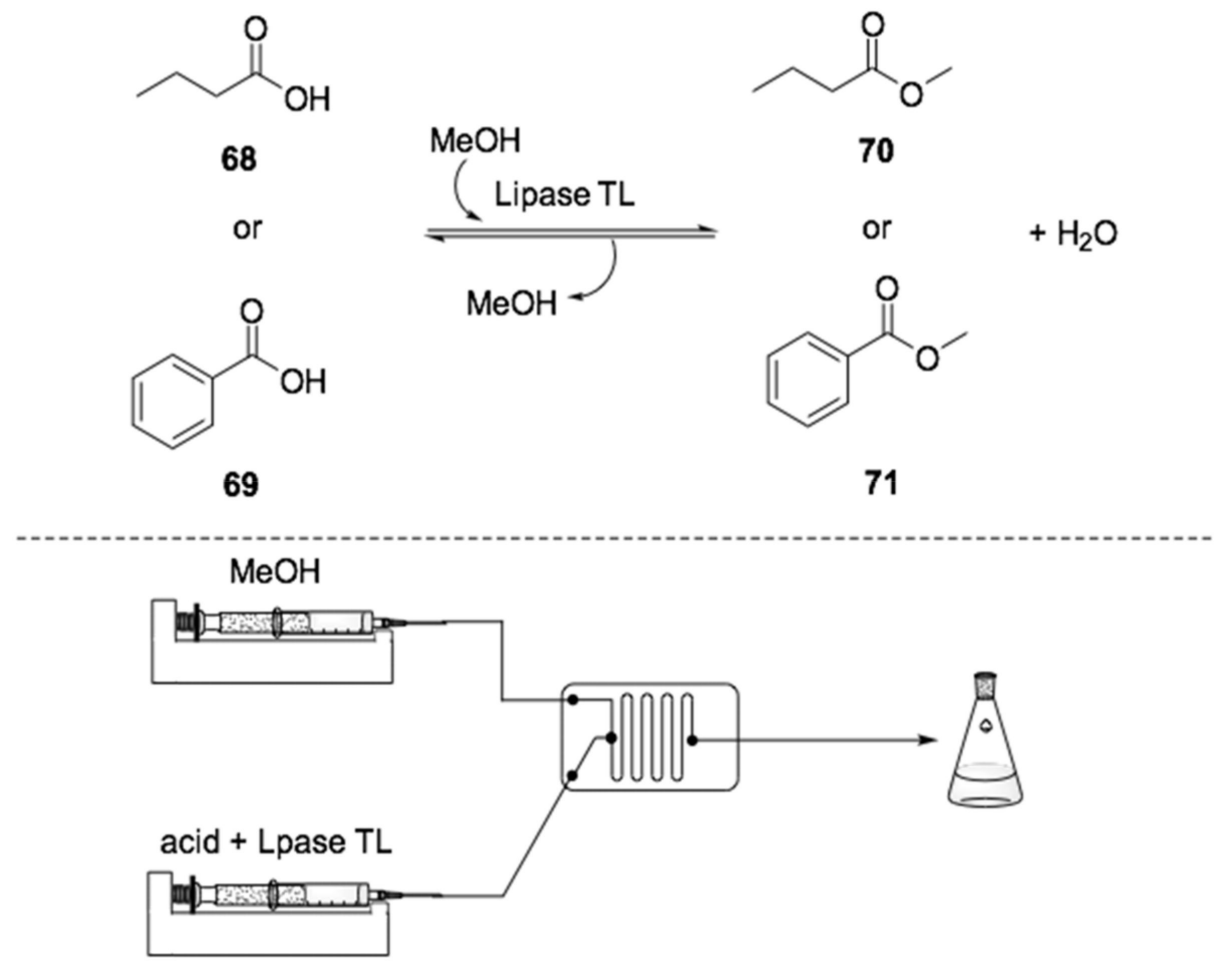
Schematic overview of a microreactor setup for the flow biocatalyzed synthesis of esters **70**–**71** [152].

In 2016, Gumel and coworkers employed the enzyme *Thermomyces lanuginosus* (lipase TL) as a catalyst in an esterification reaction [152]. Through the use of a microreactor, this methodology was applied to the synthesis of the flavor esters methyl benzoate (**70**) and methyl butanoate (**71**, Scheme 20). In this case, a solution containing the corresponding carboxylic acids **68**–**69**, along with the lipase TL in dichloromethane, was pumped together with pure methanol into a microreactor. The reactants were mixed at a flow rate of 0.1 mL·min^−1^ at 40 °C, and the system afforded the desired esters in good yields, avoiding the formation of side products.

**Scheme 21:**
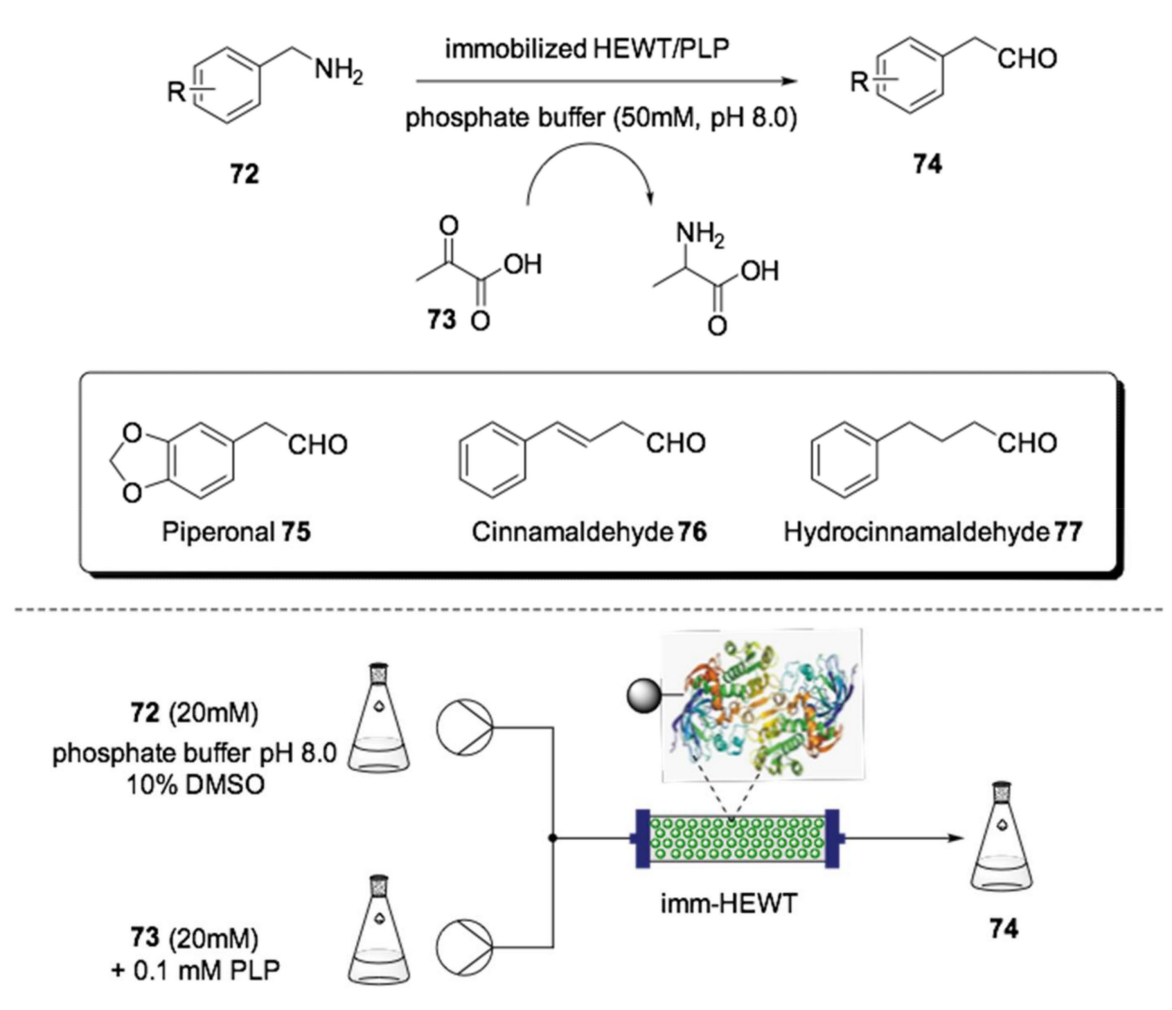
Schematic overview for the flow biocatalyzed synthesis of aldehydes **75**–**77** [153].

Aromatic aldehydes are a class of organic compounds that are mostly employed as fragrance and flavor components. In 2017, the Paradisi group reported the environmentally benign biocatalytic conversion of amines **72** to aldehydes **74** using a PBR (Scheme 21) [153]. The reaction was catalyzed by the immobilized amine transaminase from the bacterium *Halomonas elongata* (HEWT) in a phosphate buffer of pH 8.0. Pyruvic acid (**73**) was also used as an acceptor of the amine group, generating the amino acid l-alanine as a natural and easily recoverable byproduct. The products were obtained at conversion rates up to 99% in remarkably short times, ranging from 3 to 15 min, depending on the substrate. This methodology allowed the preparation of valuable aldehydes, such as piperonal **75** (used as violet fragrance), cinnamaldehyde **76** (used as cinnamon aroma) and hydrocinnamaldehyde **77** (used as honey aroma).

**Scheme 22:**
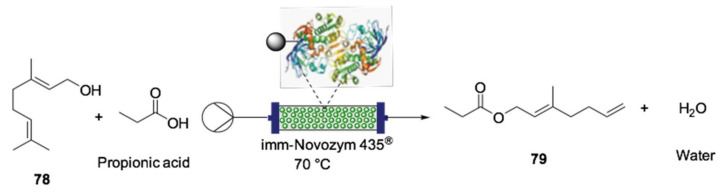
Flow biocatalyzed synthesis of industrially interesting aroma [156].

As previously mentioned, terpenes are valuable compounds that are widely abundant in nature and present interesting bioactive or agrochemical properties. Additionally, monoterpene esters are valuable compounds due to their organoleptic properties, having considerable industrial application in the preparation of flavorings. With the growing demand for the use of sustainable chemical processes, biocatalysis associated with flow chemistry has been employed with this purpose as it allows greater selectivity, energy efficiency and recyclability of the enzyme [154,155]. For instance, geraniol (**78**) is a valuable terpene found in diverse essential oils derived from geraniums. The pleasant rose-like aroma makes this natural product widely used in the synthesis of fragrances. The esterification of geraniol provides industrially important molecules with flavor, fragrance, food and pharmaceutical applications. In this regard, Salvi et al. prepared a series of geranyl esters using a continuous-flow PBR containing immobilized *Candida antarctica* lipase B (Novozym 435^®^) [156]. By this method, the geranyl propionate (**79**) was obtained with 88% conversion in 15 min from geraniol and propionic acid at a 1:1 mol ratio (Scheme 22). Moreover, the biocatalyst could be regenerated and reused for seven cycles with minimum loss of activity.

**Scheme 23:**
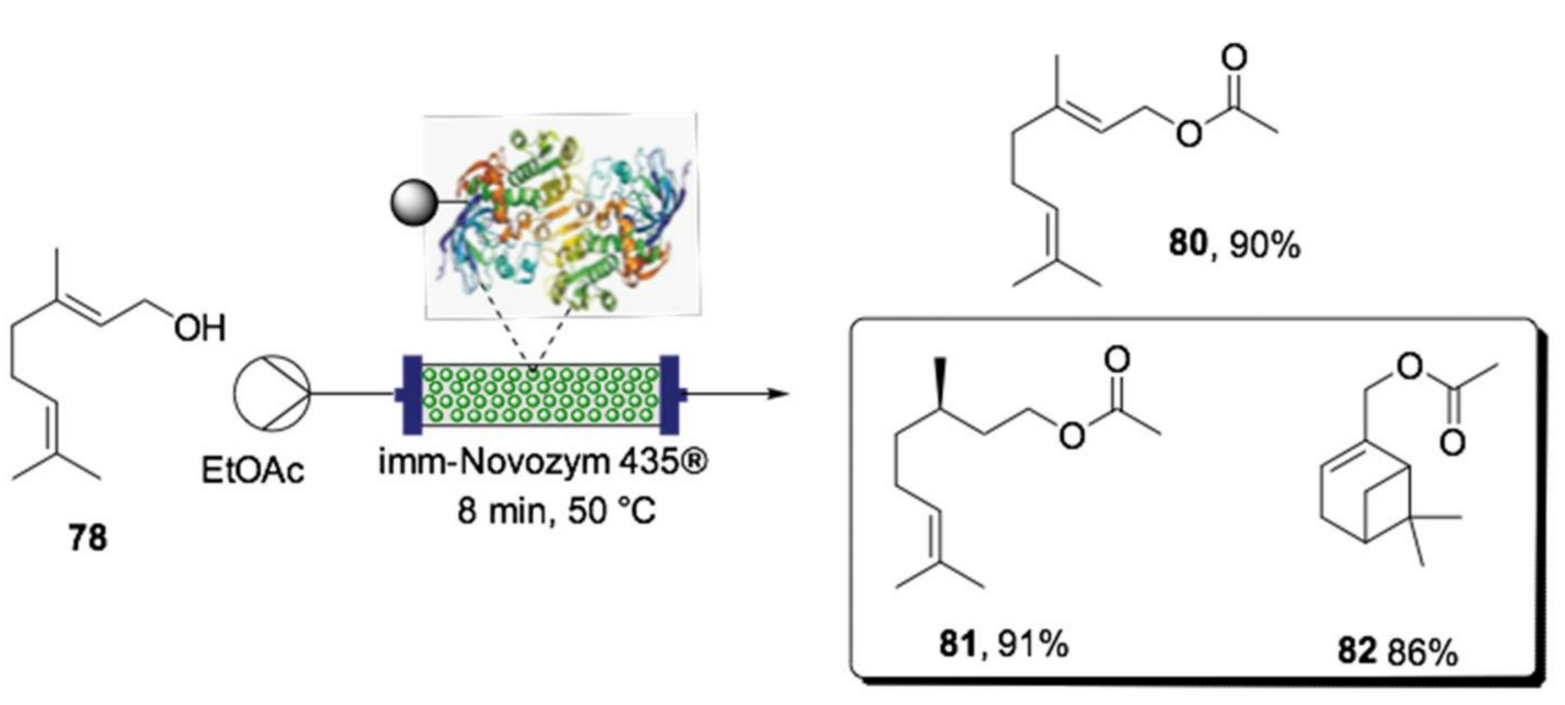
Flow biocatalyzed preparation of acetylated esters **80**–**82** [157].

In a similar approach, immobilized Novozym 435^®^ was used as biocatalyst in the flow transesterification of monoterpene alcohols in the presence of ethyl acetate as an acylating agent in a PBR [157]. The developed methodology allowed the preparation of valuable esters, such as acetylated geraniol (**80**), citronellol (**81**) and myrtenol (**82**), as well as essential oil from *Cymbopogon martini*, in high conversions at 50 °C and within only 8 min of residence time (Scheme 23). Furthermore, the authors also extended this protocol to the flow biosynthesis of monoterpene esters by using acetic anhydride as an acylating agent in the absence of a catalyst and achieved high conversions in short times.

**Scheme 24:**
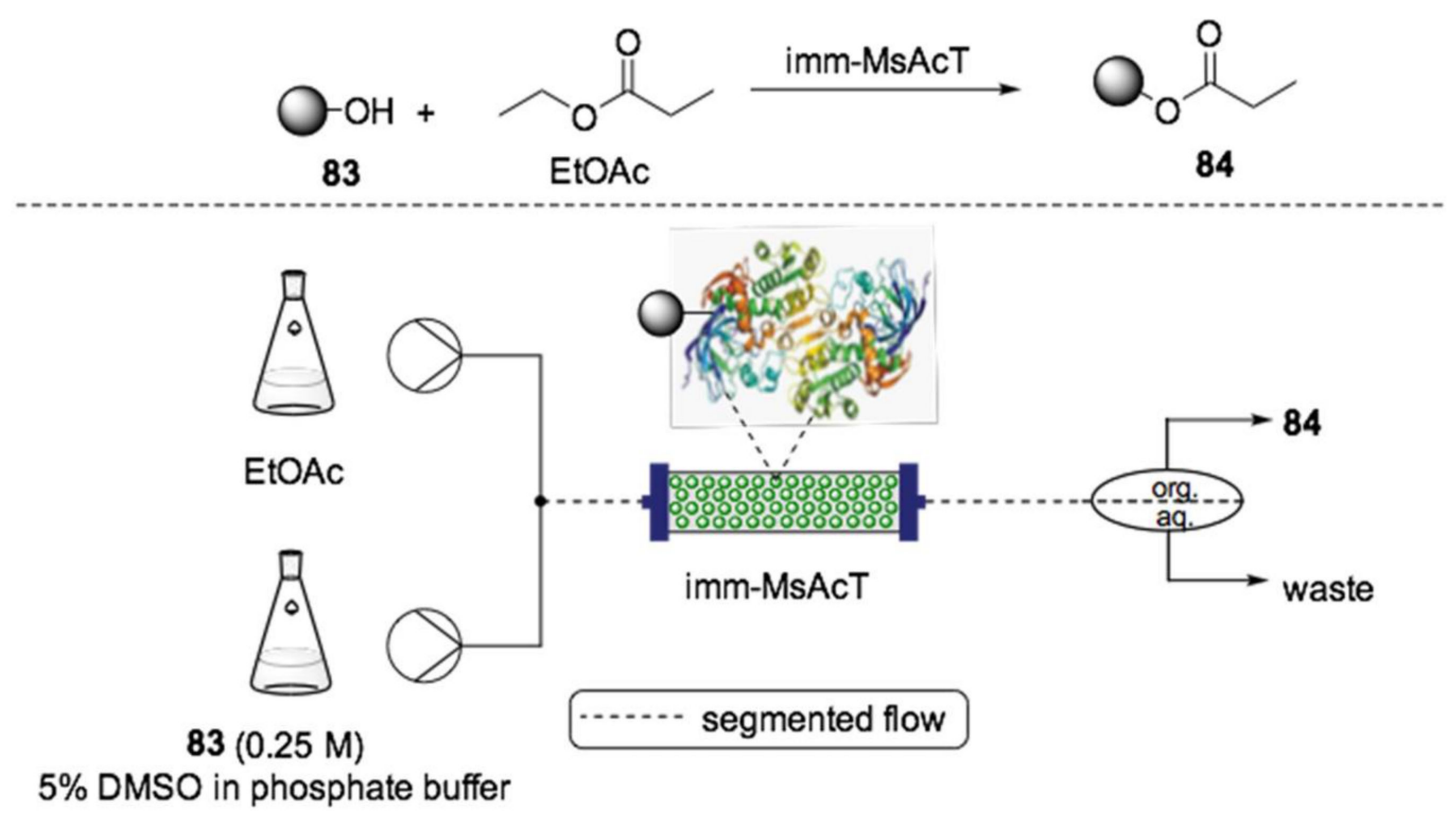
Flow biocatalyzed preparation of acetylated esters (**84**) [158].

Recently, the same group applied flow biocatalysis to the synthesis of several flavor esters by transesterification reaction (Scheme 24) [158]. The reaction of natural alcohols **83** and ethyl acetate as a solvent and acyl donor was carried out in a PBR using immobilized transferase from *Mycobacterium smegmatis* (MsAcT). A large number of esters **84** with commercial value could be obtained in up to 90% of conversion in only 5 min reaction time, which demonstrated the efficiency of the developed method.

## 6. Conclusions

A review of the most recent literature evidenced that the use of biocatalysts in continuous flow systems could represent an efficient, green and sustainable alternative for industrial processes in the synthesis of APIs and pharmaceutically-relevant synthons. The use of biocatalysis often simplifies the preparation of compounds by reducing the number of steps, which can avoid byproduct formation and increase the overall final yields. Furthermore, continuous setups overcome some typical issues connected to the use of enzymes as catalysts, increasing their stability and recyclability, simplifying the workups and enabling more efficient and less expensive scale-up and process intensification.

While the strict regulations of the FDA regarding changes to manufacturing processes are slowing down the implementation of novel continuous processes in industrial production, the general interest in this research field is growing. More efficient and reliable flow biocatalytic systems are currently being designed, developed and optimized. Thus, we believe that a remarkable shift towards more sustainable synthetic strategies could be registered in the near future, including in the pharmaceutical environment. Nevertheless, considering that some of the technologies reported in this work are still in the stage of academic investigation, it is too early to fully discuss their advantages and disadvantages regarding concrete scalability to industrial API production requirements. Hence, certain aspects, such as method validation, reproducibility and qualification, may still be challenging at this time.

## Data Availability

Data sharing not applicable.

## Abbreviations

ACE	Angiotensin-converting enzyme
AceKMs	*Mycobacterium smegmatis* acetate kinase
ADS	Amorphadiene synthases
AhPNP	*Aeromonas hydrophila* purine nucleoside phosphorylase
API	Active pharmaceutical ingredient
araA	Arabinofuranosyl adenine
araU	Arabinofuranosyl uracil
AS	Aristolochene synthase
BPR	Back pressure regulator
BS2m	*Bacillus subtilis* esterase
CCC	Counter current chromatography
CpUP	*Clostridium perfrigens* uridine phosphorylase
CLEASs	Crosslinked enzyme aggregates
CLECs	Crosslinked enzyme crystals
CSTR	Continuously stirred tank reactor
DHAA	Dihydroartemisinic aldehyde
DHAP	Dihydroxyacetone phosphate
DoE	Design of experiments
dUrd	Deoxyuridine
EMA	European Medicines Agency
FDA	Food and Drug Administration
FDP	Farnesyl diphosphate
FdUrd	5-fluoro-2′-deoxyuridine
FEP	Fluorinated ethylene propylene
FruA	Fructose aldolase
G3PD Ec	*Escherichia coli* glycerol-3-phosphate dehydrogenase
Gd4olS	Germacradien-4-ol synthase
Gd11olS	Germacradien-11-ol synthase
GlpKTk	*Thermococcus kodakarensis* glycerol kinase
Gs-Lys6DH	*Geobacillus stearothermophilus* dehydrogenase
HEWT	*Halomonas Elongata* amine transaminase
ID	Internal diameter
IdUrd	5-Iodo-2′-deoxyuridine
IMERs	Immobilized enzyme reactors
HCCC	High-performance countercurrent chromatography
HTS	High-throughput screening
LrNDT	*Lactobacillus reuteri* nucleoside 2′deoxyribosyltransferase
MsAcT	*Mycobacterium smegmatis* acyltransferase enzyme
MTBE	Methyl *tert*-butyl ether
NOXCa	NADH oxidase *Clostridium aminovalericum*
NSAID	Nonsteroidal anti-inflammatory drug
P5C	Pyrroline-5-carboxylate reductase
PBR	Packed-bed reactor
PTFE	Polytetrafluoroethylene
QbD	Quality by design
TTL	*Thermomyces lanuginosus* lipase
TTN	Total turnover number
